# A CRISPR-Cas9-mediated F0 screen to identify pro-regenerative genes in the zebrafish retinal pigment epithelium

**DOI:** 10.1038/s41598-023-29046-5

**Published:** 2023-02-23

**Authors:** Fangfang Lu, Lyndsay L. Leach, Jeffrey M. Gross

**Affiliations:** 1 Department of Ophthalmology, University of Pittsburgh School of Medicine, Pittsburgh, PA 15213 USA; 2 Department of Ophthalmology, The Second Xiangya Hospital, Central South University, Changsha, 410011 Hunan China; 3 Department of Molecular Biosciences, The University of Texas at Austin, Austin, TX 78712 USA

**Keywords:** Regeneration and repair in the nervous system, Macular degeneration

## Abstract

Ocular diseases resulting in death of the retinal pigment epithelium (RPE) lead to vision loss and blindness. There are currently no FDA-approved strategies to restore damaged RPE cells. Stimulating intrinsic regenerative responses within damaged tissues has gained traction as a possible mechanism for tissue repair. Zebrafish possess remarkable regenerative abilities, including within the RPE; however, our understanding of the underlying mechanisms remains limited. Here, we conducted an F0 in vivo CRISPR-Cas9-mediated screen of 27 candidate RPE regeneration genes. The screen involved injection of a ribonucleoprotein complex containing three highly mutagenic guide RNAs per target gene followed by PCR-based genotyping to identify large intragenic deletions and MATLAB-based automated quantification of RPE regeneration. Through this F0 screening pipeline, eight positive and seven negative regulators of RPE regeneration were identified. Further characterization of one candidate, *cldn7b*, revealed novel roles in regulating macrophage/microglia infiltration after RPE injury and in clearing RPE/pigment debris during late-phase RPE regeneration. Taken together, these data support the utility of targeted F0 screens for validating pro-regenerative factors and reveal novel factors that could regulate regenerative responses within the zebrafish RPE.

## Introduction

The retinal pigment epithelium (RPE) is a monolayer of cells that function to support vision^1^. RPE dysfunction contributes to many ocular degenerative diseases, including age-related macular degeneration^2^. Mammals have a limited ability to repair RPE damage and therefore RPE dysfunction results in permanent visual impairment^3,4^. An intriguing therapeutic approach to overcome irreversible RPE damage is to leverage the intrinsic regenerative potential of RPE in situ^5–9^. For this to become a reality, we must understand the mechanisms driving intrinsic RPE regeneration. Zebrafish possess robust regenerative competence and share genetic synteny with humans^10^. Thus, knowledge gained from identifying mechanisms driving RPE regeneration in zebrafish can be applied to non-regenerative mammals, including humans. We previously demonstrated an intrinsic RPE regenerative capacity in zebrafish^11^. Subsequent experiments identified an extensive list of potential RPE regeneration regulators, including mTOR^12^ and immune^13^ pathway responses. Many candidate genes of interest (GOIs) remain to be investigated in vivo and higher-throughput methodology is needed for GOI screening.

Clustered regularly interspaced short palindromic repeats (CRISPR)-Cas9 technology has emerged as a facile system for generating genetic mutations^14–17^. This utility is due to the simplicity of designing single guide RNAs (sgRNAs), the efficiency and precision of targeting virtually any genomic locus, and the ease of multiplex genome editing^18,19^. Indeed, CRISPR-Cas9 technology has been widely applied in zebrafish to systematically investigate gene function^20,21^. Historically, however, phenotypes have often been analyzed in F2 generations, when homozygous mutants are obtained^22,23^, limiting throughput. F0 screens have emerged as a rapid method for identifying phenotypes, but their efficacy depends on the mutagenic efficiency of CRISPR-Cas9. Recent improvements to targeted F0 mutagenesis include multiplexed pooled injections of gRNAs^24^, use of pre-assembled Cas9-sgRNA ribonucleoprotein complexes (RNPs)^25,26^, use of synthetic two-component CRISPR RNA and trans-acting CRISPR RNA (crRNA:tracrRNA) guide RNAs (gRNAs)^27^, and multi-locus targeting^27–30^. Of interest here, Kroll et al. maximized CRISPR-Cas9 editing efficiency by combining multi-locus targeting with methodology to enhance mutagenesis at each locus^29^. Targeting three unique loci per gene with synthetic gRNAs consistently converted > 90% of injected embryos directly into biallelic knockouts. Additionally, headloop PCR^31^ was adapted to rapidly validate the mutagenic activity of individual gRNAs. The high proportion of biallelic knockouts in F0 populations coupled with economical PCR-based mutagenic screening make this an excellent approach for high-throughput screens.

Here, we selected 27 RPE regeneration GOIs identified from previous RNA-seq studies^12,13^ and combined our zebrafish RPE ablation-regeneration model^11^ with CRISPR-Cas9-mediated F0 knockout technology to develop a scalable F0 screening pipeline to identify essential regulators of RPE regeneration. This pipeline is composed of the Kroll et al. F0 knockout methodology^29^, PCR amplification-based genotyping, and a MATLAB-based automated quantification platform, RpEGEN^32^. Eight potential positive regulators and seven potential negative regulators of RPE regeneration were identified in the screen. Further characterization of one GOI, *cldn7b*, revealed an essential role in modulating the activity of macrophages/microglia during late-phase RPE regeneration.

## Results and discussion

### A CRISPR/Cas9-based F0 screening pipeline to identify genes that modulate RPE regeneration

**Figure 1:**
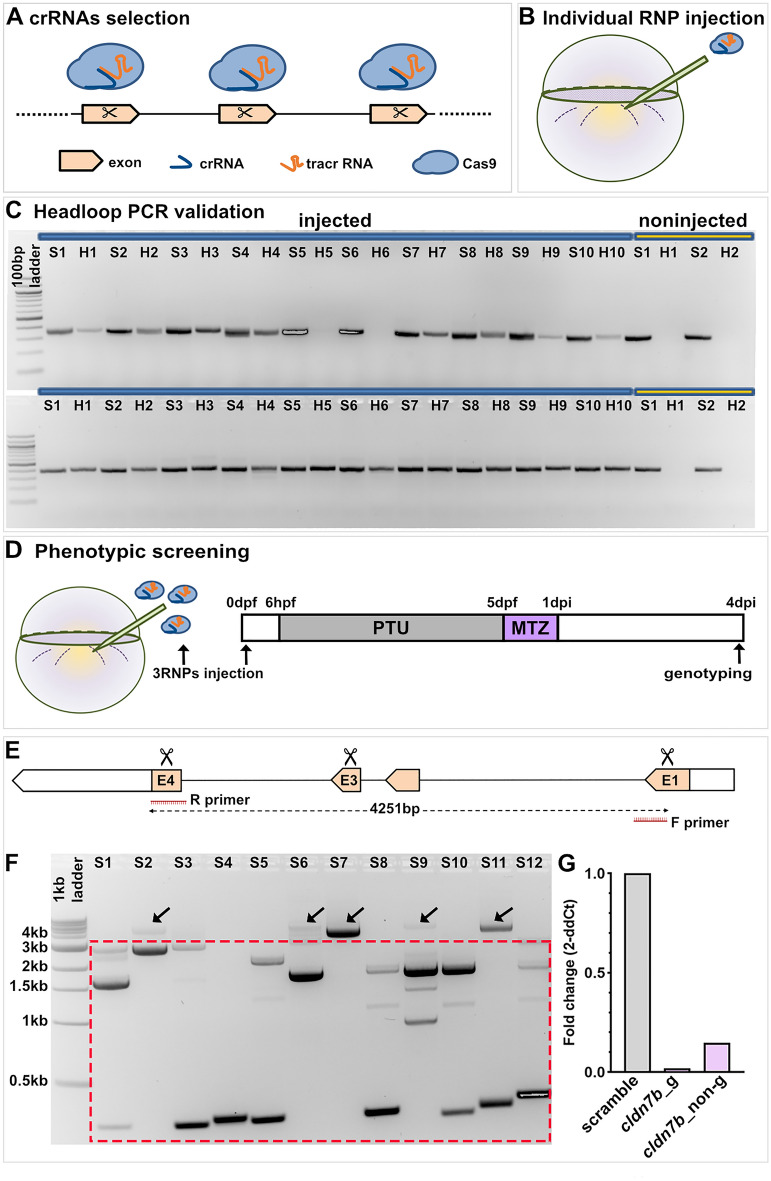
F0 screening pipeline to identify genes regulating RPE regeneration. (**A**) Design and selection of crRNAs. (**B**) Injection of individual RNP complexes (gRNAs (crRNA:tracrRNA) with Cas9 protein) into *rpe65a*:nfsB-eGFP embryos. (**C**) Example electrophoresis results of headloop PCR (H) targeting *zgc:*153911 (top gel) and *nrg1* (bottom gel) in parallel with standard PCR (S) from ten RNP-injected embryos and two uninjected controls. Mutagenic rate = H+/S+ number of embryos. Mutagenic rate for *zgc:*153911: 80%; for *nrg1*: 100%. (**D**) Schematic of the injection/ablation and screening paradigm. (**E**) Schematic of *cldn7b* showing exon 1 (E1), 3 (E3), and 4 (E4) target loci (scissors) and PCR strategy to confirm intragenic deletions. (**F**) Example *cldn7b* data from individual RNP-injected larvae confirming intragenic deletions generated by simultaneously targeting three genomic sites. Gel bands within the dashed box indicate intragenic deletions. ~ 4 kb bands (arrows) represent either wild-type or mutated alleles with small indels. (**G**) Bar graph showing relative expression fold change of *cldn7b* from genotyped (*cldn7b*_g) and non-genotyped (*cldn7b*_non-g) F0 knockout larvae and scrambled controls. Each bar represents biological variation from n = 50 pooled larvae and technical variation from three replicates. Example gels shown in (**C**) and (**F**) have been color inverted and cropped for clarity and data presentation purposes. Original gel images are shown in Fig. S9. Abbreviations as follows: RNP, ribonucleoprotein; PTU, n-phenylthiourea; MTZ, metronidazole; hpf/dpf, hour/day (s) post-fertilization; dpi, day (s) post-injury.

Recent RNA-seq data from our laboratory have identified numerous factors with the potential to regulate RPE regeneration^12,13^. Here, we sought to develop a pipeline to rapidly screen and validate GOIs for roles in RPE regeneration. For the screen, we selected 27 GOIs from amongst the top 100 genes upregulated in RPE cells at 2 days post-injury (dpi) (Fig. S1)^12,13^ based on predicted functions (e.g. cell–cell contact, lipid metabolism, immune responses) that are critical during tissue regeneration in a variety of organs/systems^33,34^. To generate knockouts, we modified an existing CRISPR/Cas9-based F0 mutagenesis methodology (Fig. 1A–D)^29^. For each GOI, three crRNAs with Alt-R modifications were designed, each of which targeted non-overlapping regions of the 5′ exons (Fig. 1A). RNP complexes were preassembled in vitro using Cas9 protein and dual (crRNA:tracrRNA) gRNAs^27^. Headloop PCR^31^ was used to pre-screen individual RNP complexes to ensure substantial mutagenesis at each locus (Fig. 1B,C). As described by Kroll et al. this technique suppresses amplification of wild-type sequences, producing no PCR product, but effectively amplifies sequences with indels^29^. For example, headloop PCR validation of *zgc:*153911 RNPs revealed two embryos lacking headloop PCR bands, indicating the presence of the wild-type sequence and failed mutagenesis (Fig. 1C; top gel, lanes H5 and H6). The mutagenic efficiency of each RNP was determined quantitatively, by assessing the ratio of headloop PCR bands (H), which indicate an effective mutagenesis event, to standard PCR bands (S); and qualitatively, by comparing H and S band intensities (Fig. 1C). In the case of *zgc:*153911, eight of the ten embryos screened showed successful mutagenesis (Fig. 1C; top gel, H lanes) yielding a mutagenic ratio of 80%. gRNAs showing a mutagenic ratio > 70% were used for phenotypic screening (Table S1).

After confirming effective mutagenesis of individual RNPs, a pool of three RNPs per GOI was injected into one-cell stage embryos, while control embryos were injected with a pool of three scrambled RNPs predicted not to target the zebrafish genome (Fig. 1D). Regardless of the gene targeted, pooled RNP injections resulted in dead and/or dysmorphic embryos, with incidences ranging from 13.3 to 40.5% (Table S1), consistent with published results^29^; these dysmorphic embryos were removed from the study. For all GOIs tested for potential roles in RPE regeneration, F0 knockout and scrambled control larvae assayed showed no overt developmental RPE phenotypes. To confirm biallelic mutations in F0 knockout larvae used for phenotypic analysis, we performed PCR amplification of sequences spanning all three GOI target sites, with the expectation that large intragenic deletions caused by the joining of sequences distal to the single RNP-induced double-strand breaks would be detectable (Fig. 1E). Indeed, large deletions were detected in most injected embryos (Fig. 1F). These likely represent loss-of-function mutations that prevent the expression of the protein of interest^27,35^ and/or functional rescue through genetic compensation^36^. For 20 of the 27 GOIs, only genotyped F0 larvae carrying large intragenic deletions were used for phenotypic analyses. For the remaining seven GOIs, it was not feasible to PCR amplify across target sites due to the size of the locus (Table S1). Therefore, while the individual RNPs were each validated for these seven GOIs, the combined efficiency of the three pooled RNPs was not assessed. To further validate our F0 knockout pipeline, we used quantitative real-time PCR (qRT-PCR) to measure mRNA levels of one GOI, *cldn7b,* in genotyped F0 knockout larvae. As anticipated, qRT-PCR results showed a ~ 98% decrease in *cldn7b* expression in genotyped *cldn7b* F0 knockout larvae with large deletions, when compared to scrambled controls (Fig. 1G). Moreover, pooled/non-genotyped *cldn7b* F0 knockout larvae showed a ~ 85% loss of *cldn7b* expression when compared to scrambled controls, suggesting that the pooled RNPs were highly effective in mutagenizing the target locus and reducing gene expression, even when large intragenic deletions were not pre-screened/enriched. Taken together, these results establish an efficient and robust F0 screening pipeline to identify RPE regeneration factors.

### Phenotypic screening identified essential regulators of RPE regeneration

**Figure 2:**
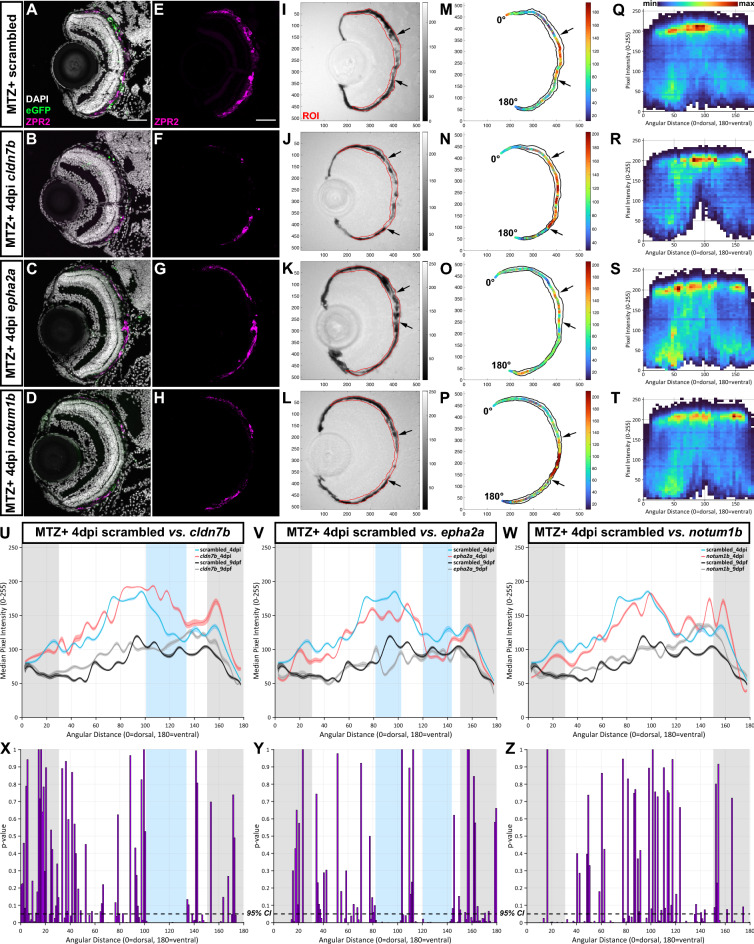
Representative screening results. (**A**–**L**) Representative immunofluorescence images of transverse cryosections from ablated (MTZ^+^) scrambled, *cldn7b, epha2a,* and *notum1b* RNP-injected F0 larvae at 4dpi. Single-channel ZPR2 (**E**–**H**) and brightfield (**I**–**L**) images. Grayscale bars indicate an 8-bit scale (0 = black, 255 = white). Black arrows delineate edges of pigment recovery. Regions of interest (ROIs) are outlined in red. Nuclei (white), eGFP (green), ZPR2 (magenta). (**M**–**P**) Median pixel intensity distributions in the ROI. Color bars indicate an 8-bit scale (0 = dark blue, 255 = dark red). (**Q**–**T**) Heatmaps showing dorsal-to-ventral (angular distance from 0 to 180°) distribution of raw pixel intensity values within each ROI from ablated scrambled, *cldn7b, epha2a,* and *notum1b* RNP-injected F0 larvae at 4dpi. Color bar above (**Q**) indicates bin counts. (**U**–**W**) Plots showing median ROI pixel intensity values from 4dpi ablated (blue and red lines) and 9dpf unablated (black and gray lines) scrambled, *cldn7b, epha2a,* and *notum1b* RNP-injected F0 larvae. (**Q**–**W**) Bin size = 5 angular degrees. (**X**–**Z**) Statistical comparisons of dorsal-to-ventral median pixel intensity between: (**X**) scrambled and *cldn7b,* (**Y**) scrambled and *epha2a*, and (**Z**) scrambled and *notum1b*. Blue shading indicates regions with significant differences spanning > 20 angular degrees. Gray shading indicates the dorsal (0 to 30 angular degrees) and ventral (150 to 180 angular degrees) peripheral RPE areas omitted from analyses. Dashed black lines indicate a 95% confidence interval (CI), Bin size = 1 angular degree. Scale bars = 50 μm. Exact regions with significant differences compared to scrambled controls can be found in Table S2Abbreviations as follows: *MTZ* Metronidazole; *dpi* Days post-injury; *min* Minimum; *max* Maximum.

For phenotypic analysis, nitroreductase/metronidazole (NTR/MTZ)-mediated ablation of the RPE was conducted on F0 knockout larvae and scrambled controls carrying the *rpe65a*:nfsB-eGFP transgene (Fig. 1D). RPE markers (e.g., ZPR2) and pigment are largely recovered by 4dpi in control larvae^11–13^ and here, we used these readouts to assess the impact of GOI knockout on RPE regeneration at 4dpi. To facilitate large-scale phenotyping, quantification of normalized brightfield images was performed using RpEGEN, a MATLAB-based platform developed to automate quantification of RPE/pigment recovery^32^. RpEGEN quantifies 8-bit pixel intensities within an RPE region of interest (ROI), which extends 0 to 180 angular degrees (dorsal to ventral), and statistically compares results between GOI and scrambled controls. Here, the same group of scrambled controls (n = 16 larvae from 3 independent injections) was used for individual GOI comparisons. RPE regions spanning 0–30 angular degrees (distal-most dorsal RPE) and 150–180 angular degrees (distal-most ventral RPE) were omitted, as *rpe65a*:nfsB-eGFP expression and MTZ-induced ablation are confined to the central two-thirds of the RPE^11^. To identify phenotypes, we established a selection criterion in which continuous blocks of statistically significant pigment differences (*p-*value ≤ 0.05) spanning > 20 angular degrees would be considered phenotypically relevant. Using this criterion, eight GOIs *(cldn7b, fosl1b, nrg1, ogflr1, ccn1l1, lipib, cidec,* and *il11a)* showed angular degree block(s) with significantly higher median pixel intensity (indicating lighter pixels/less pigmentation) in F0 knockout larvae, when compared to scrambled controls (Fig. 2U,X, Fig. S2, Table S2). These GOIs were determined to be positive regulators of RPE regeneration. Seven genes *(cpa4, zgc:*153911*, adamtsl7, epha2a, dkk1a, lepb,* and *serpine1)* showed angular degree blocks with significantly lower median pixel intensity (indicating darker pixels/more pigmentation) in F0 knockout larvae, when compared to scrambled controls (Fig. 2V,Y, Fig. S3, Table S2). These GOIs were determined to be negative regulators of RPE regeneration. The remaining 12 genes did not satisfy the criterion defined here for a phenotype (Fig. 2W,Z, Fig. S4). Figure 2 shows a representative example from each group.

While it was determined that 12 GOIs were not essential regulators of RPE regeneration, this assessment was made using pigment recovery as a screening criterion and does not take into consideration other aspects of RPE regeneration such as the recovery of apical/basal polarity or functionality of the regenerated RPE. It is likely that using a series of integrated phenotypic screens applied in parallel to the same pool of F0 knockout larvae would further expand this list of RPE regeneration regulators. F0 screens using pre-screened RNPs have been shown to result in high phenotypic penetrance and diversity of null alleles with > 80% of alleles harboring a frameshift mutation^29^. Here, of the seven GOIs for which we could not amplify across potential intragenic deletions, five showed regeneration phenotypes. Thus, while pre-screening RNPs for large deletion competency is helpful to ensure screened embryos possess a mutagenized locus, this is not an absolute requirement for phenotype detection.

The representative positive regulator, *cldn7b*, showed a noticeable expansion of the central RPE injury site, which lacked ZPR2 signal (Fig. 2B,F) and pigmentation (Fig. 2J) in F0 knockout larvae when compared to scrambled controls (Fig. 2A,E,I). RpEGEN quantitation showed an extended distribution of lighter pixels with higher raw (Fig. 2R) and median (Fig. 2N) intensity values in the RPE of *cldn7b* F0 knockout larvae when compared to scrambled controls (Fig. 2M,Q). Dorsal-to-ventral RPE pigment quantification revealed significantly higher median pixel intensity (indicating less pigmentation) from 101 to 133 angular degrees in the *cldn7b* F0 knockout group, when compared to scrambled controls (Fig. 2U,X). By comparison, the representative negative regulator, *epha2a*, showed both ZPR2 distribution (Fig. 2C,G) and pigment recovery (Fig. 2K) extended further into the central RPE injury site in F0 knockout larvae when compared to scrambled controls (Fig. 2A,E,I). Consistently, *epha2a* F0 knockout larvae also showed an overall distribution of darker pixels with lower intensity values (Fig. 2O,S), and there were blocks of significant median pixel intensity differences from 82 to 102 and 120–143 angular degrees in *epha2a* F0 knockout larvae compared to scrambled controls (Fig. 2V,Y). Finally, *notum1b* F0 knockout larvae, representative of the “no phenotype” group, showed similar ZPR2 distribution (Fig. 2D,H) and pigment recovery (Fig. 2L) to that of scrambled controls (Fig. 2A,E,I; arrows). Quantification of median pixel intensity values in *notum1b* F0 knockout larvae (Fig. 2P,T) did not reveal significant differences with scrambled controls (Fig. 2M,Q,W,Z). Broadly, unablated (MTZ−) 9dpf F0 knockout larvae did not display any overt RPE phenotypes when examined histologically. As an example, structural integrity of the RPE-photoreceptor (PR) interface appeared intact in unablated (MTZ−) *cldn7b* F0 knockout larvae at 9dpf when compared to scrambled controls (Fig. S5). Indeed, PRs showed normal lamination (Fig. S5B’,D’; red asterisks) and RPE were polarized with basally localized nuclei (Fig. S5B’,D’; arrows as examples) and prominent apical microvilli (Fig. S5B”,B’’’,D’’,D’’’; arrowheads as examples) in both larval groups. Further, there were no differences between unablated (MTZ−) 9dpf *cldn7b, epha2a,* and *notum1b* RpEGEN plots (Fig. 2U–W; gray lines) when compared to scrambled controls (Fig. 2U–W; black lines).

### Macrophages/microglia are retained in the RPE layer of F0 *cldn7b* knockout larvae during regeneration

To further validate the robustness of our F0 screen, we characterized the requirement for *cldn7b* during RPE regeneration. Claudin-7b is a multifunctional protein that maintains apical tight junction (TJ) barrier integrity^37^ and epithelial cell polarity^38^ in zebrafish. Claudin-7 also functions in suppressing cell proliferation and migration by modulating cell–matrix interactions^39–41^. Multiple studies using *claudin-7* knockout mice have also highlighted its role in regulating inflammatory responses^42–44^. As proliferation occurs rapidly post-RPE ablation^11^, we first explored whether *cldn7b* knockout impacted cell proliferation in regenerating RPE. As anticipated, the number of proliferating (BrdU+) cells in the RPE layer was significantly increased in 4dpi ablated (MTZ+) scrambled (Fig. S6C) and *cldn7b* knockout (Fig. S6D) larvae when compared to respective 9dpf unablated (MTZ−) controls (Fig. S6A,B; quantified in Fig. S6E). However, there was no significant difference in numbers of BrdU+ cells between *cldn7b* F0 knockouts and scrambled controls in either unablated (MTZ−) or ablated (MTZ+) groups (Fig. S6E), indicating cldn7b does not contribute to the proliferative response post-injury.

**Figure 3:**
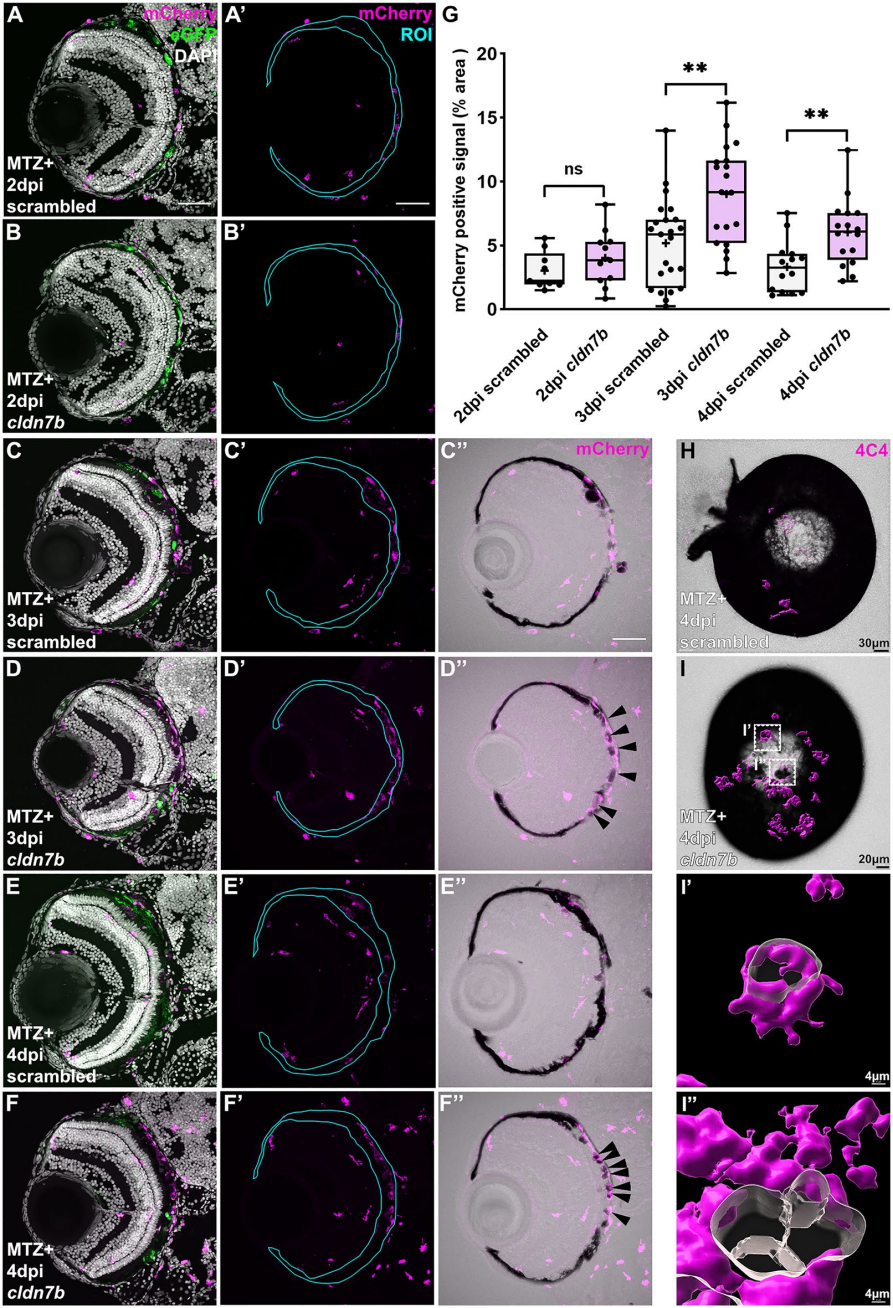
*cldn7b* F0 larvae show retention of macrophages/microglia and impaired clearance of pigment debris during RPE regeneration. (**A**–**F**) Representative immunofluorescence images of mCherry signal in ablated (MTZ +) scrambled and *cldn7b* F0 larvae at (**A**,**B**) 2dpi, (**C**,**D**) 3dpi, and (**E**,**F**) 4dpi. (**A’**–**F’**) Single-channel images of mCherry signal in RPE ROIs. (**C’’**–**F’’**) Overlay of mCherry and bright-field channels. Nuclei (white), eGFP (green), mCherry (magenta). (**G**) Box plots showing significantly more mCherry+ macrophage/microglia in the RPE layer of *cldn7b* F0 larvae at 3dpi and 4dpi, compared to scrambled controls. (**H**,**I**) Overlaid images of 3D-rendered 4C4+ isosurfaces (magenta) with brightfield channels from ablated scrambled and *cldn7b* knockout whole-mount eyes. 4C4+ macrophages/microglia (magenta) surfaces were 3D-rendered in Imaris. (**I’**, **I’’**) Zoom-in of (I; white boxes) *cldn7b* F0 whole-mount eye showing 4C4+ cells engulfing pigment debris. (**A**–**F**) Scale bars = 50 μm. (**H**–**I**) Scale bars were indicated on the images. **p value ≤ 0.01; *ns* not significant. Exact *p* values, numbers of independent experiments (N), and numbers of biological replicates (n) can be found in Table S3. Abbreviations as follows: *MTZ* Metronidazole; *dpi* Days post-injury; *ROI* Region of interest.

Innate immunity and inflammation play a critical role during RPE regeneration^13^. In response to injury, macrophages/microglia accumulate in/around the RPE between 2 and 3dpi, likely acting as scavengers to phagocytose apoptotic bodies and cell debris, and triggering an inflammatory response. Between 3 and 4dpi, macrophage/microglia accumulation wanes and inflammation likely resolves to facilitate recovery of pigment/RPE^13^. With this in mind, we next sought to investigate whether *cldn7b* knockout affects RPE regeneration by modulating injury-induced immune responses. To test this hypothesis, we utilized *mpeg1*:mCherry zebrafish^45^ to assess macrophage/microglia localization from 2 to 4dpi. At 2dpi, macrophages/microglia accumulated in the RPE injury site, but there was no significant difference between ablated *cldn7b* F0 knockout larvae and scrambled controls (Fig. 3A,A’,B,B’,G). By 3dpi, substantial infiltration of mCherry+ macrophages/microglia was observed in the RPE layer in both *cldn7b* F0 knockout larvae and scrambled controls (Fig. 3C, C’,D,D’), as anticipated^13^, and quantification showed significantly more macrophages/microglia in ablated *cldn7b* F0 knockout larvae relative to scrambled controls (Fig. 3G). Overlaying mCherry and brightfield channels revealed a close association between mCherry+ cells and pigment debris (Fig. 3C’’,D’’), which was especially evident in *cldn7b* F0 knockout larvae where the mCherry+ signal appeared to encompass pigment clusters (Fig. 3D’’). By 4dpi, macrophage/microglia presence waned in scrambled controls (Fig. 3E,E’,H,G), while significant retention of mCherry+ signal was observed in the injury site of *cldn7b* F0 knockout larvae (Fig. 3F,F’,I,G). Notably, there were no significant differences in mCherry signal quantification between the unablated groups at any time point (Fig. S7G–I), nor were there any significant changes in TUNEL staining in *cldn7b* F0 knockout larvae at 3dpi (Fig. S8A–E) and 4dpi (Fig. S8F–J) that would account for increased macrophage/microglia accumulation. These observations are consistent with those in *Cldn7* knockout mice where loss of Cldn7 promoted leukocyte infiltration and intestinal inflammation by damaging the intestinal epithelium^46^, increasing intestinal epithelial permeability, and disrupting cell–matrix interactions^42–44^. Collectively, these data indicate a role for *cldn7b* in regulating leukocyte infiltration in response to RPE injury and suggest that increased macrophages/microglia accumulation in the RPE layer post-injury might reflect non-TJ functions of claudin-7 in modulating cell–matrix adhesion.

Pigment was largely recovered in control larvae at 4dpi (Fig. 3E’’), as anticipated^11^, whereas *cldn7b* F0 knockout larvae showed less pigment recovery and the remaining pigment debris appeared to be surrounded by macrophages/microglia (Fig. 3F’’). 3D-rendering of whole-mount eyes labeled with 4C4, a marker for zebrafish macrophages/microglia^47,48^, further highlighted the accumulation of macrophages/microglia in *cldn7b* F0 knockout eyes (Fig. 3H,I). Moreover, the 4C4+ signal surrounded pigment debris (Fig. 3I-I”), suggesting a possible role for claudin-7b in assisting macrophage/microglia with phagocytosis/removal of degenerating RPE post-ablation.

The expression of claudins is species-specific, tissue-specific, and often cell-specific or stage-specific during development. Our *cldn7b* F0 knockout model cannot distinguish whether cldn7b is required in the RPE, macrophages/microglia, and/or other cells (like RPE stem cells)^49^ involved in regeneration. Claudin expression/distribution is not known in zebrafish RPE, but in cultured human fetal RPE, claudin-19 and claudin-3 are enriched^50^, while in murine RPE, *Cldn1, Cldn2, Cldn4, Cldn7,* and *Cldn23* are all expressed^51^. Macrophages also express claudin-1, claudin-2, claudin-11 and claudin-7^52,53^. Claudin-1 in macrophages is predicted to interact with the airway epithelium and help preserve tissue integrity^54^. Claudin-1 is also thought to aid pathogen clearance by macrophages in an in vitro intestinal epithelium model^55^. Phagocytosis of apoptotic cells by macrophages facilitates expression of anti-inflammatory markers and the switch to an anti-inflammatory phenotype^56,57^. cldn7b deficiency in zebrafish could impair the resolution of inflammation during the later phases of RPE regeneration and thereby impede regenerative processes.

In summary, we report an effective and rapid F0 screening platform to identify regulators of RPE regeneration in zebrafish. From a pilot screen, we identified 15 novel RPE regeneration regulators, the further study of which will greatly expand our understanding of regenerative processes. Some of the candidates might be useful foundations around which therapeutics could be developed to slow or even treat RPE degenerative diseases.

## Methods

### Ethics statement

All animal experiments were performed in accordance with relevant guidelines and regulations and with approval from the University of Pittsburgh School of Medicine Institutional Animal Care and Use Committee. Methods are also reported in accordance with ARRIVE guidelines.

### Zebrafish husbandry

Adult zebrafish *(Danio rerio)* were maintained under standard conditions at 28.5 °C in circulating system water on a 14-h light/10-h dark cycle. Embryos and larvae (≤ 9dpf) used for subsequent experiments were kept in the dark in an incubator at 28.5 °C until being euthanized by tricaine overdose (MS-222; Western Chemical Inc.).

### RPE ablation

RPE ablation was performed using the transgenic *rpe65a:*nfsB-eGFP zebrafish line (mw86Tg) as described using an established protocol^32^ Briefly, embryos derived from wide-type AB and *rpe65a:*nfsB-eGFP outcrosses or *mpeg1:*mCherry (gl23Tg)^45^ and *rpe65a:*nfsB-eGFP outcrosses were treated with 0.003% 1-phenyl 2-thiourea (PTU; Sigma-Aldrich) from 6hpf to 5dpf to prevent pigmentation. At 5dpf, larvae were screened for the *rpe65a:*nfsB-eGFP and/or *mpeg1:*mCherry transgenes and immersed in 10 mM metronidazole (MTZ; Sigma-Aldrich) dissolved in system water for RPE ablation. MTZ was removed after 24-h treatment and fresh system water was replenished daily until larvae were euthanized for experiments.

### TUNEL and BrdU incorporation assays

For bromodeoxyuridine (BrdU) incorporation experiments, larvae were exposed to system water containing 10 mM BrdU (Sigma-Aldrich) for 24 h before euthanasia. TUNEL experiments were performed on 12 µm sections from tissues embedded in optimal cutting temperature (OCT) using the Invitrogen Click-iT Plus TUNEL Assay Kits for In Situ Apoptosis Detection (Alexa Fluor 647 dye, ThermoFisher Scientific). The manufacturer protocol for tissue sections was followed without deparaffinization and with one modification: 0.5X (1:50) Proteinase K solution was used for tissue permeabilization. Nuclei were counterstained using 1 mg/ml DAPI (1:250, Sigma-Aldrich) according to manufacturer instructions.

### crRNA selection/design and RNP preparation and injection

crRNAs for each target gene were either selected from the Alt-R Predesigned Cas9 crRNA Selection Tool or designed using the Alt-R Custom Cas9 crRNA Design Tool within the Integrated DNA Technologies (IDT) database. crRNAs were selected based on published criteria^29^ and designed to target three distinct and asymmetric exons shared by all/most transcripts for the target genes. RNPs were prepared as described^29^; briefly, the crRNA was annealed with an equal molar amount of tracrRNA (IDT, #1072532) in duplex buffer (IDT, #11010301) to form gRNA by heating at 95 °C for 5 min and subsequently cooling on ice. gRNA was assembled with an equal molar amount of Alt-R S.p. Cas9 Nuclease V3 (IDT, #1081058) to form the RNP complex (28.5 µM final concentration) by incubation at 37 °C for 5 min followed by storage at − 20 °C. For pre-screening validation of individual RNPs, approximately 1 nl of the single RNP complex (28.5 µM final concentration) was injected into the yolk of one-cell stage embryos before cell inflation. For phenotypic screening, three RNPs (9.5 µM each individual RNP) were pooled to yield a final concentration of 28.5 µM RNPs (or 4700 pg Cas9 and 1000 pg each gRNA per embryo). Approximately 1 nl of the three-RNP complex pool was injected into one-cell stage embryos. To account for any non-specific effects resulting from the injection itself, pooled RNP complexes were prepared from three scrambled crRNAs (IDT: Alt-R CRISPR-Cas9 Negative Control crRNA #1, 1072544; #2, 1072545; #3, 1072546) and injected as described above. GOIs targeted showed no overt developmental RPE phenotypes in F0 knockout or scrambled control larvae. Additional details about the design method, target locus, and sequence of each crRNA can be found in Table S1.

### Standard, headloop, and genotyping PCR

Genomic DNA was extracted from embryos or larval tails by incubating with 50ul of 50 mM NaOH at 95 °C for 20 min. After cooling to 4 °C, 5ul of 1 M Tris–HCl (pH 8) was added for neutralization. For standard PCR, each 25 µl reaction contained 1 µl of genomic DNA, 1 µl 10 µM forward and reverse primer mix, 0.5 µl 10 mM dNTPs, 5 µl standard 5X Phusion HF Buffer, and 0.1 µl of Phusion DNA polymerase (NEB). For headloop PCR, one of the primers was replaced by a 2.5 µM primer tagged with 20 bases that are completely complementary to the target locus. Standard and headloop PCR products from n = 10 RNP-injected embryos and n = 2 uninjected embryos were run in parallel on a 1% agarose gel for data analysis. For genotyping PCR, the same reaction as standard PCR was used to amplify a short (< 1 kb) sequence spanning all three target sites. For amplicons > 1 kb, 25ul reactions were modified to include: 2 µl of genomic DNA, 2.5 µl 10 µM primer mix, 0.5 µl 10 mM dNTPs, 5 µl standard 5X Phusion HF Buffer, and 0.2 µl of Phusion DNA polymerase. Longer amplicons were analyzed on a 2% agarose gel. Additional details about primers used in standard, headloop, and genotyping PCR can be found in Table S1.

### Quantitative real-time PCR (qRT-PCR)

Total RNA was extracted and purified from pooled scrambled control and genotyped *cldn7b* F0 knockout larvae (n = 50 per group) at 7dpf using the RNeasy Plus Micro Kit (QIAGEN), followed by cDNA synthesis with the SuperScript IV VILO Master Mix Kit (ThermoFisher). Forward (5′-AATCCTCTCTGTTGGAGCCCT-3′) and reverse (5′-TTGACAGGTGTGAAGGGGTTG-3′) primers spanning the *cldn7b* exon 2-exon 3 junction were designed using Primer BLAST (https://www.ncbi.nlm.nih.gov/tools/primer-blast/). Each qRT-PCR reaction (10 µl/well) was assembled by adding 2 µl 0.5 ng/µl cDNA, 0.5 µl 10 µM forward and reverse primer mix, and 5 µl 2X iTaq Universal SYBR Green Supermix (Bio-Rad Laboratories). Experiments were run in three technical replicates on a CFX384 Touch Real-Time PCR Detection System (Bio-Rad Laboratories). Relative gene expression fold change was calculated using the Livak method (2^−ΔΔCt^)^58^. GAPDH was the housekeeping gene chosen for expression normalization^59^ as *cldn7b* knockout did not affect baseline expression.

### Immunohistochemistry on cryosections and whole-mount larvae

Larvae were fixed in 4% paraformaldehyde (PFA) at room temperature for 2–3 h or overnight at 4 °C. Immunostaining on cryosections was performed following an established protocol^60^. Briefly, fixed larvae were rinsed in phosphate-buffered saline (PBS) twice, followed by cryoprotection with sucrose (25% to 35% gradient). Subsequently, tissues were embedded in OCT compound and rapidly frozen on dry ice. Transverse cryosections were cut at 12-micron thickness and mounted onto poly-L-lysine pre-coated glass slides (Superfrost Plus; Thermo Fisher). For mCherry and ZPR2 staining, slides were rehydrated in PBS and washed three times with PBTD (1X PBS with 0.1% Tween-20 and 1% DMSO). For BrdU staining, an antigen retrieval step, which consisted of incubating sections in 4N HCl for 8 min at 37 °C, was added before washes in PBTD. Then, sections were blocked for ≥ 2 h in 5% normal goat serum (NGS) in PBTD (e.g. blocking buffer) before overnight incubation at 4 °C with primary antibody diluted in blocking buffer. Sections were subsequently washed three times with PBTD and incubated for 2–3 h with secondary antibody diluted in blocking buffer. Slides were then washed in PBTD for 3 × 10 min and 1 mg/ml DAPI (1:250) was added and incubated for 10 min between the first and second washes. Slides were mounted with Vectashield mounting media (Vector Laboratories) and sealed with nail polish.

Whole-mount staining was performed as described^61^. Briefly, fixed tissues were rinsed with PBST (PBS with 0.1% Tween-20) and distilled water, followed by permeabilization with 100% acetone at − 20 °C for 10 min and subsequent rinsing with distilled water and PBST. Tissues were then digested in a collagenase solution (1 mg/mL in PBST) for 30 min and followed by proteinase K (2 mg/mL in PBST) exposure for another 30 min before blocking for ≥ 1 h blocking in 2% NGS in PBDTX (PBS with 1% bovine serum albumin, 1% DMSO, and 0.1% Triton X-100, pH = 7.3). Larvae were then incubated overnight at 4 °C with primary antibody diluted in blocking buffer. The next day, larvae were washed with PBDTX four times and incubated for 4 h at room temperature in secondary antibody diluted in blocking buffer. Nuclei were counterstained with DAPI, which was added for the last 2 h of the 4-h secondary incubation. Subsequently, larvae were rinsed three times in PBS. Eyes were dissected using sharpened tungsten wire and mounted onto slides with 0.5% low melting agarose immediately before imaging. Primary antibodies used in this study include: mouse anti-zpr-2 (1:250, Zebrafish International Resource Center (ZIRC)), mouse anti-mCherry (1:200, Takara Bio/Clontech Laboratories, 632543), rat anti-BrdU (1:250, Abcam, ab6326), and mouse anti-4C4 (1:200, a kind gift of Dr. Peter Hitchcock, University of Michigan School of Medicine, USA). Secondary antibodies used in this study include goat anti-mouse Cy3 (1:250, Jackson ImmunoResearch Laboratories, 115-165-166) and goat anti-rat Cy3 (1:250, Jackson ImmunoResearch Laboratories, 112-165-003). Fluorescent images for TUNEL experiments were acquired on a Nikon A1R confocal microscope (Nikon Instruments, Inc.) at the Center for Biomedical Research Support Microscopy and Imaging Facility at UT Austin (RRID# SCR_021756). All other fluorescent images were captured using an Olympus Fluoview FV1200 laser scanning confocal microscope (Olympus Corporation). Cryosection images were taken under 40 × objectives (Olympus Corporation and Nikon Instruments, Inc.) and whole-mount images were taken under a 20 × objective with 1.7 × zoom (Olympus Corporation).

### Imaging processing and quantification

Quantification using FIJI (ImageJ^62^): Raw confocal z-stack images (1 µm z-step size) were processed for image presentation and quantification as described^12^. To quantify BrdU and TUNEL, z-stack images were maximum-projected and the number of BrdU + cells or TUNEL + puncta in the RPE layer were counted manually using the Cell Counter Plugin. Each data point represents the average number of BrdU + cells or total number (summation) of TUNEL + puncta from three consecutive central sections per larva. To quantify mCherry signal, maximum-projected images were first converted into 8-bit TIF images. Using the Polygon Selection Tool, RPE regions of interest (ROIs) were drawn to encompass the space between the outer edge of the photoreceptor outer segments (e.g. the apical RPE/photoreceptor interface) and the basal-most edges of RPE pigment. Thresholding on the mCherry channel was then performed to measure the percent area of mCherry+ signal within the ROI. The same thresholding parameters (40/255, 8-bit scale) were applied to all images to standardize quantification. Each data point represents the quantification of a single, central-most section from each larva.

Quantification using MATLAB: For ablated (MTZ+) 4dpi larvae, raw confocal z-stack images (1 µm z-step size) were obtained from n = 16 scrambled larvae from 3 independent injections and at least n = 8 larvae from genotyped F0 GOI knockout larvae. Images from n = 6 scrambled (3 independent injections) and at least n = 4 genotyped F0 GOI knockout unablated (MTZ−) 9dpf sibling controls were obtained to assess any overt RPE phenotype. Images with poor tissue quality and/or RPE tearing were excluded from analysis in MATLAB to avoid misrepresentation of pigment quantification. Images were preprocessed into 8-bit grayscale TIFs and RPE ROIs were drawn using FIJI, as described in detail^32^. Image normalization was achieved by first averaging the mean gray values of each brightfield image in the whole dataset (e.g. all scramble control and GOI images), which averaged to 196 (0–255 8-bit scale). Then, the Brightness Tool (FIJI) was used to adjust the mean gray value of each individual brightfield image to 196/255. Subsequently, normalized TIF images and ROI files were processed in MATLAB (R2021b (version 9.11.0) using the RpEGEN scripts, as described in detail^32^. Robust statistical comparisons between scrambled controls and F0 knockout groups were validated and achieved using 20,000 permutation simulations.

### Statistics

All statistical analyses were performed using Prism 9.0 (GraphPad). For box plots, the line and plus within the box represent the median and mean, respectively; the top and bottom whiskers represent the maximum and minimum values, respectively; and each dot represents a biological replicate (one eye from one larva). For statistical tests, D’Agostino-Pearson omnibus normality test was first performed to determine whether data obeyed normal (Gaussian) distributions. For comparisons between two groups, unpaired Student’s t-tests with Welch’s correction were performed on datasets with normal distribution and non-parametric Mann–Whitney tests were performed on datasets that were not normally distributed. For multiple comparisons, Kruskal–Wallis ANOVA followed by Dunn's multiple comparisons tests were used to determine the significance between groups. Exact *p* values, numbers of independent experiments (N), and numbers of biological replicates (n) for each dataset can be found in Table S3.

## Supplementary Information

Supplementary Information.

## Data Availability

RNA-seq datasets used for this study are publicly available through NCBI GEO: Series GSE174538^12^ and Series GSE155294^13^. The RpEGEN scripts used for quantification in this study are freely available and can be downloaded from the GitHub repository: https://github.com/burchfisher/RpEGEN^32^.
