# Supplementary material for: A CRISPR-Cas9-mediated F0 screen to identify pro-regenerative genes in the zebrafish retinal pigment epithelium
Source: Sci Rep. 2023 Feb 23;13:3142. doi: 10.1038/s41598-023-29046-5 (PMC9950062; doi:10.1038/s41598-023-29046-5)
Supplement: Supplementary file 1 — Supplementary Information. [file 41598_2023_29046_MOESM1_ESM.pdf]

SUPPLEMENTARY INFORMATION

Fig. S1. Relative gene expression of 27 genes of interest selected for the phenotypic screen.

Relative gene expression (log_2 fold change) of 27 significant (FDR *p*-value <0.05) differentially expressed genes (DEGs) selected from top upregulated genes in RPE cells at 2 days post-injury (dpi) compared to 7 days post-fertilization (dpf) from a publicly available bulk RNA sequencing dataset (GSE174538; Lu, F., Leach, L. L. & Gross, J. M. mTOR activity is essential for retinal pigment epithelium regeneration in zebrafish. *PLoS Genet.* **18**, e1009628 (2022)).

Fig. S2. Phenotypic screening results of additional positive regulators of RPE regeneration.

(A-G) RpEGEN output plots showing median pixel intensity distributions (bin size = 5 angular degrees) within RPE regions of interest (ROIs) and statistical comparisons of dorsal-to-ventral median pixel intensity (bin size = 1 angular degree) between: (A) scrambled and *nrg1* groups, (B) scrambled and *fosl1b* groups, (C) scrambled and *ogflr1* groups, (D) scrambled and *ccn1l1* groups, (E) scrambled and *lipib* groups, (F) scrambled and *cidec* groups, and (G) scrambled and *il11a* groups. All plots represent comparisons between larval groups at 4dpi. Light blue rectangles indicate the region(s) with significantly higher (lighter) pixel intensity values spanning more than twenty angular degrees. Gray rectangles indicate the distal-most dorsal (0 to 30 angular degrees) and distal-most ventral (150 to 180 angular degrees) peripheral RPE areas omitted from analyses. Dashed black lines indicate a 95% confidence interval (CI). Exact regions with significant differences compared to scrambled controls can be found in Table S2. Abbreviation: dpi, days post-injury.

Fig. S3. Phenotypic screening results of additional negative regulators of RPE regeneration.

(A-F) RpEGEN output plots showing median pixel intensity distributions (bin size = 5 angular degrees) within RPE regions of interest (ROIs) and statistical comparisons of dorsal-to-ventral median pixel intensity (bin size = 1 angular degree) between: (A) scrambled and *zgc:153911* groups, (B) scrambled and *cpa4* groups, (C) scrambled and *adamtsl7* groups, (D) scrambled and *dkk1a* groups, (E) scrambled and *lepb* groups, and (F) scrambled and *serpine1* groups. All plots represent comparisons between larval groups at 4dpi. Light blue rectangles indicate the region(s) with significantly lower (darker) pixel intensity values

spanning more than twenty angular degrees. Gray rectangles indicate the distal-most dorsal (0 to 30 angular degrees) and distal-most ventral (150 to 180 angular degrees) peripheral RPE areas omitted from analyses. Dashed black lines indicate a 95% confidence interval (CI). Exact regions with significant differences compared to scrambled controls can be found in Table S2. Abbreviation: dpi, days post-injury.

Fig. S4. Phenotypic screening results of additional GOIs that did not show an RPE regeneration phenotype.

(A-J) RpEGEN output plots showing median pixel intensity distributions (bin size = 5 angular degrees) within RPE regions of interest (ROIs) and statistical comparisons of dorsal-to-ventral median pixel intensity (bin size = 1 angular degree) between: (A) scrambled and *ptx3a* groups, (B) scrambled and *ccl34a.4* groups, (C) scrambled and *clcf1* groups, (D) scrambled and *cxcl18a.1* group, (E) scrambled and *il11b* groups, (F) scrambled and *epcam* groups, (G) scrambled and *ptgs2a* groups, (H) scrambled and *ptgs2b* groups, (I) scrambled and *met* groups, (F) scrambled and *cxcl8a* groups, and (J) scrambled and *edn2* groups. All plots represent comparisons between larval groups at 4dpi. Gray rectangles indicate the distal-most dorsal (0 to 30 angular degrees) and distal-most ventral (150 to 180 angular degrees) peripheral RPE areas omitted from analyses. Dashed black lines indicate a 95% confidence interval (CI). Abbreviation: dpi, days post-injury.

Fig. S5. The RPE layer appears phenotypically normal in unablated scrambled and *cldn7b* F0 knockout larvae.

Representative immunofluorescence images of unablated (MTZ-) 9dpf (A-B’’’; n=6) scrambled and (C-D’’’; n=6) *cldn7b* F0 knockout larvae. Nuclei (white), eGFP (green), ZPR2 (magenta). (B,D) Digital zooms highlight (B’,D’; red asterisk) photoreceptor lamination, (B’,D’; cyan arrow) basal nuclei in the RPE, and distinct apical microvilli in RPE co-labeled with (B’’,D’’; white arrowhead) eGFP and (B’’’,D’’’; white arrowhead) ZPR2. Scale bars = 50μm. Abbreviations as follows: MTZ, metronidazole; dpf, days post-fertilization.

Fig. S6. *cldn7b* F0 knockout does not impact cell proliferation in the RPE layer from 3dpi to 4dpi.

(A-D) Representative immunofluorescence images showing the BrdU-labeled proliferative cells in the unablated (MTZ-) and ablated (MTZ+) scrambled and *cldn7b* F0 knockout larval eyes. Nuclei (white), BrdU (red). (E) Box plots showing significant increases in the number of BrdU-labeled cells in the RPE layer of ablated scrambled and *cldn7b* F0 knockout larvae when compared to the corresponding unablated larvae, and no significant differences of unablated or ablated *cldn7b* F0 knockout larvae when compared to scrambled controls. Scale bars = 50µm; *****P*-value ≤0.0001, ns = not significant. Exact *p*-values, numbers of independent experiments (N), and numbers of biological replicates (n) can be found in Table S3. Abbreviations as follows: BrdU, bromodeoxyuridine; MTZ, metronidazole; dpi, days post-injury.

Fig. S7. *cldn7b* F0 knockout does not affect localization of macrophages/microglia in unablated larvae.

(A-F) Representative immunofluorescence images of mCherry signals in the unablated (MTZ-) *cldn7b* F0 knockout larval eyes at (A-B') 7dpf, (C-D') 8dpf, and (E-F') 9dpf. (A'-F') Single-channel images of mCherry signal with RPE regions of interest (ROIs). Nuclei (white), eGFP (green), mCherry (magenta). (G-I) Box plots showing no significant differences in mCherry signal between scrambled and *cldn7b* knockout groups at (G) 7dpf, (H) 8dpf, and (I) 9dpf. Scale bars = 50µm; ns = not significant. Exact *p*-values, numbers of independent experiments (N), and numbers of biological replicates (n) can be found in Table S3. Abbreviations as follows: MTZ, metronidazole; dpf, days post-fertilization.

Fig. S8. *cldn7b* F0 knockout does not influence cell death in the RPE layer at 3dpi and 4dpi.

(A-D) Representative immunofluorescence images of TUNEL+ puncta in (A,B) 8dpf unablated (MTZ-) and (C,D) 3dpi ablated (MTZ+) scrambled and *cldn7b* F0 knockout larval eyes. (F-I) Representative immunofluorescence images of TUNEL+ puncta in (F,G) 9dpf unablated (MTZ-) and (H,I) 4dpi ablated (MTZ+) scrambled and *cldn7b* F0 knockout larval eyes. Nuclei (white), TUNEL (red). (E,J) Box plots showing no significant differences in TUNEL signal between scrambled and *cldn7b* knockout groups at (E) 8dpf/3dpi and (J) 9dpf/4dpi. Scale bars = 50µm; ns = not significant. Exact *p*-values, numbers of independent experiments (N), and numbers of biological replicates (n) can be found in Table S3. Abbreviations as follows: MTZ, metronidazole; dpf, days post-fertilization; dpi, days post-injury.

Fig. S9. Original images of cropped electrophoresis gels presented in Figure 1.

Original gel electrophoresis results for (A; red dotted box) *zgc:153911* headloop PCR and standard PCR shown in Fig. 1C (top gel), (B; teal dotted box) *nrg1* headloop PCR and standard PCR shown in Fig. 1C (bottom gel), and (C; blue dotted box) *cldn7b* genotyping shown in Fig. 1F.

Table S1. crRNAs and primers used in this study.

Table S2. RPE regions (>20 continuous angular degrees) with significant differences.

Table. S3. Statistics.

For box plots, the line and plus within the box represent the median and mean, respectively; the top and bottom whiskers represent the maximum and minimum values, respectively; and each dot represents a biological replicate (one eye from one larva). For statistical tests, D'Agostino-Pearson omnibus normality test was first performed to determine whether data obeyed normal (Gaussian) distributions. For comparisons between two groups, unpaired Student's t-tests with Welch's correction were performed on datasets with normal distribution and non-parametric Mann–Whitney tests were performed on datasets that were not normally distributed. For multiple comparisons, Kruskal–Wallis ANOVA followed by Dunn's multiple comparisons tests were used to determine the significance between groups. Exact *p*-values, numbers of independent experiments (N), and numbers of biological replicates (n) for each dataset can be found in Table S3.

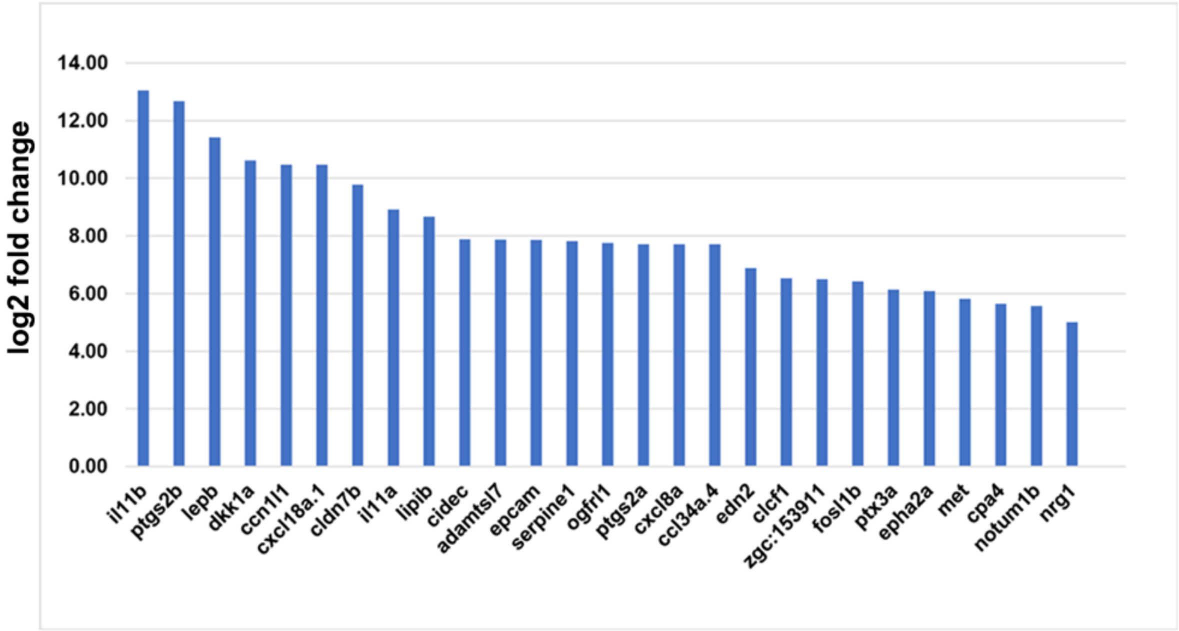
log2 fold change
14.00
12.00
10.00
8.00
6.00
4.00
2.00
0.00
il11b
ptgs2b
lepb
dkk1a
ccn1l1
cxcl18a.1
cldn7b
il11a
lipib
cidec
adamtsl7
epcam
serpine1
ogfrl1
ptgs2a
cxcl8a
ccl34a.4
edn2
clcf1
zgc:153911
fosl1b
ptx3a
epha2a
met
cpa4
notum1b
nrg1

FIGURE S1

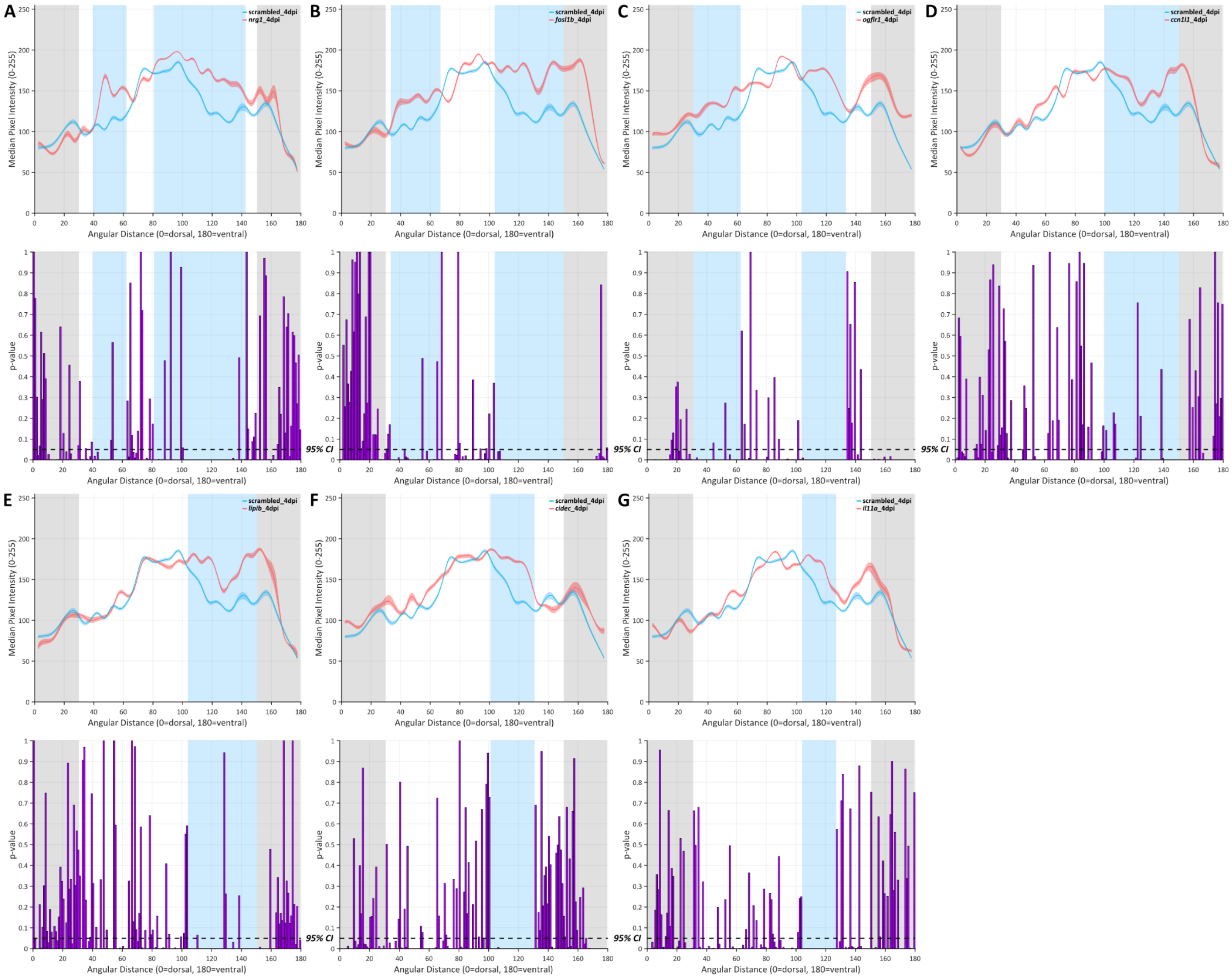
A
B
C
D
E
F
G
scrambled_4dpi
nrg1_4dpi
fosl1b_4dpi
ogflr1_4dpi
ccn1l1_4dpi
lipib_4dpi
cidec_4dpi
il11a_4dpi
Median Pixel Intensity (0-255)
Angular Distance (0=dorsal, 180=ventral)
p-value
95% CI

FIGURE S2

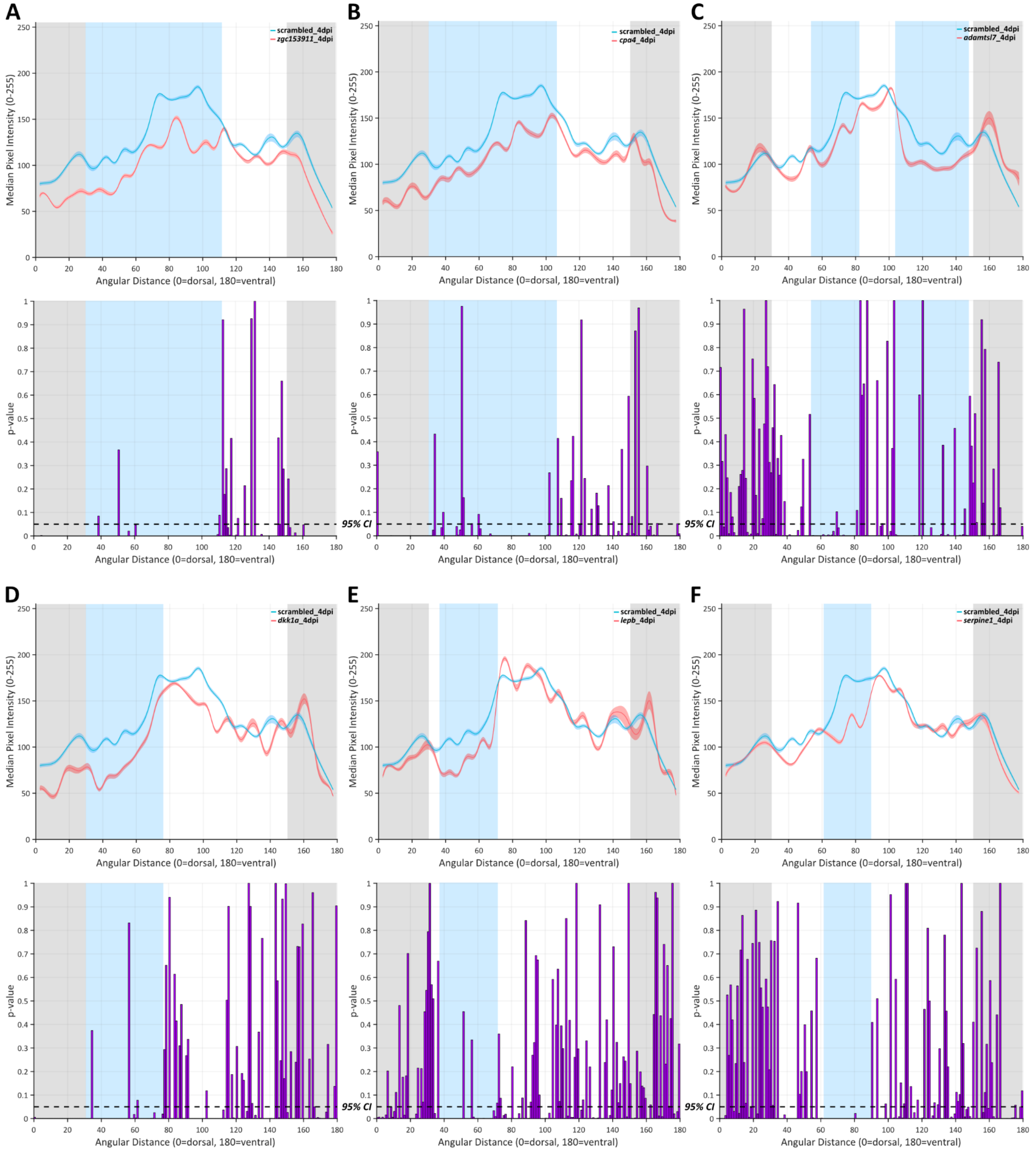
A
scrambled_4dpi
zgc153911_4dpi
Median Pixel Intensity (0-255)
Angular Distance (0=dorsal, 180=ventral)
p-value
B
scrambled_4dpi
cpa4_4dpi
95% CI
C
scrambled_4dpi
adamtsl7_4dpi
D
scrambled_4dpi
dkk1a_4dpi
E
scrambled_4dpi
lepb_4dpi
F
scrambled_4dpi
serpine1_4dpi

FIGURE S3

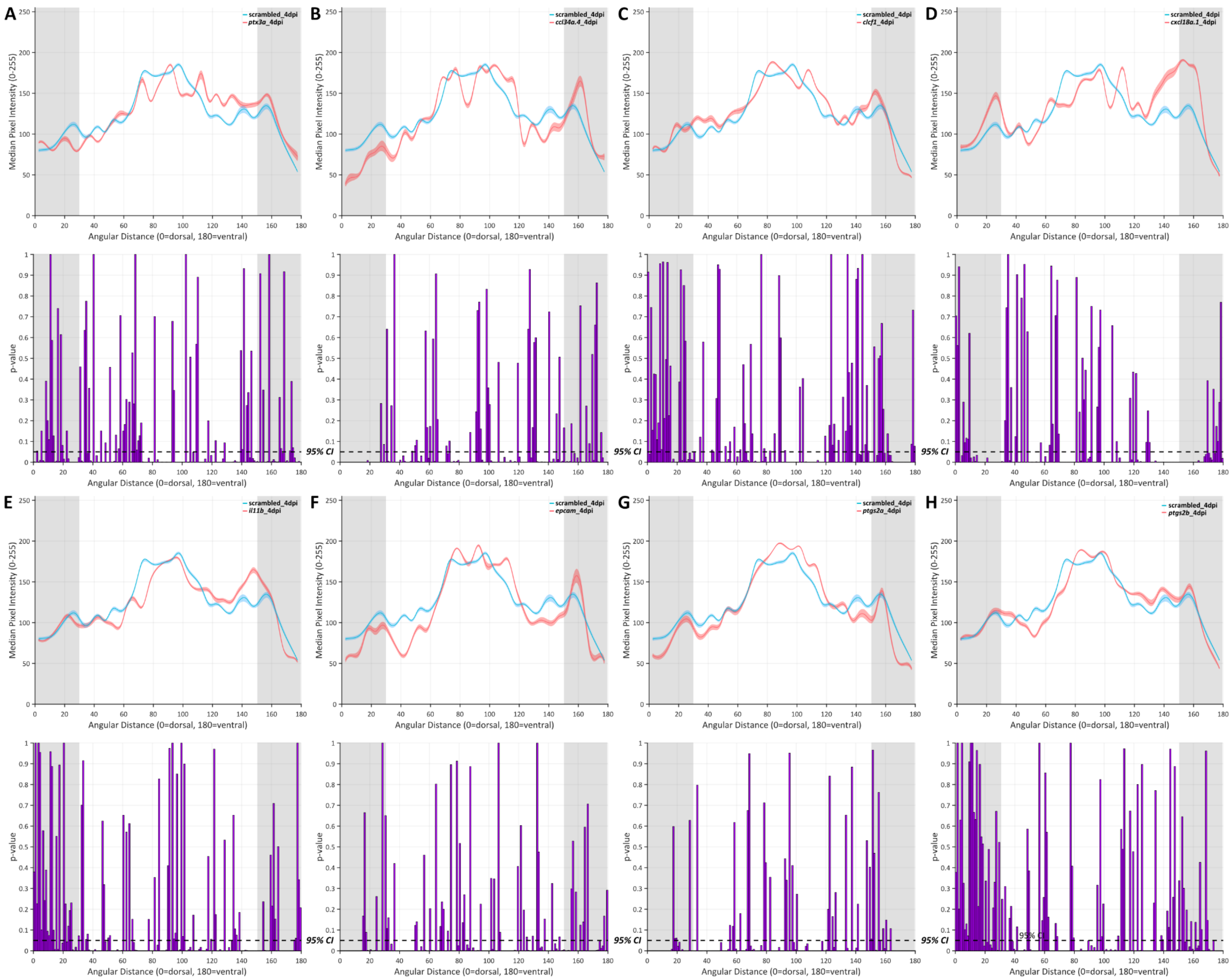

FIGURE S4 (continued on next page)

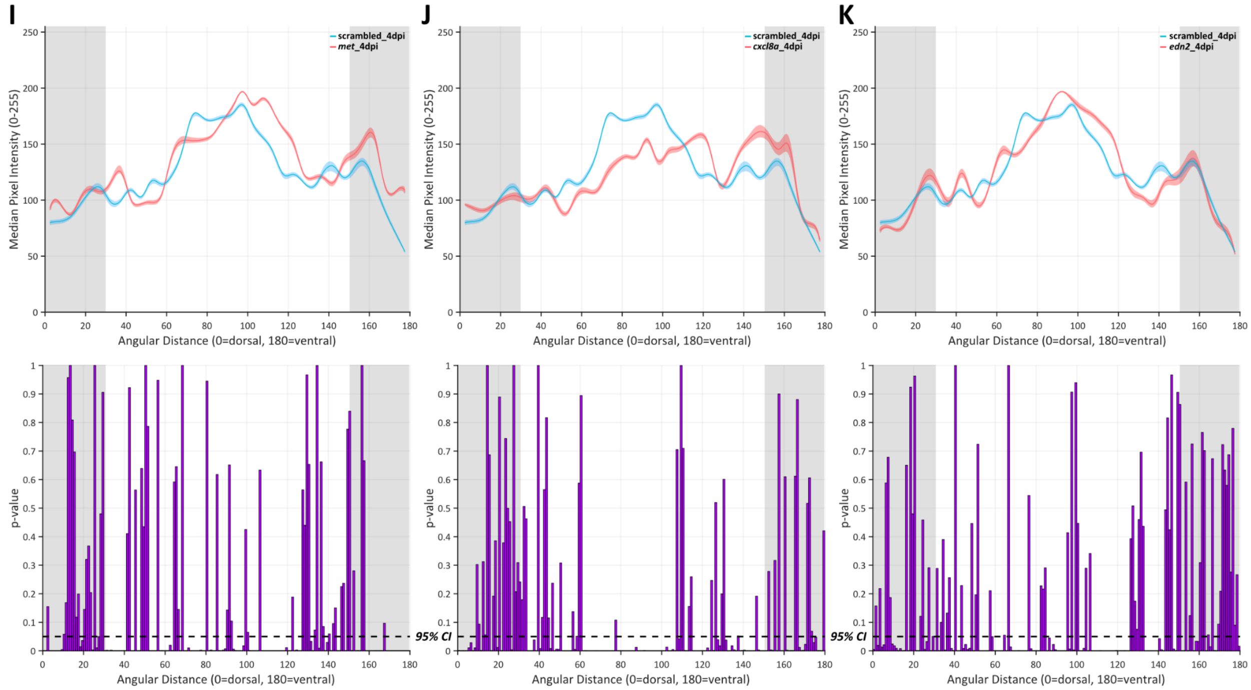

FIGURE S4 (continued from previous page)

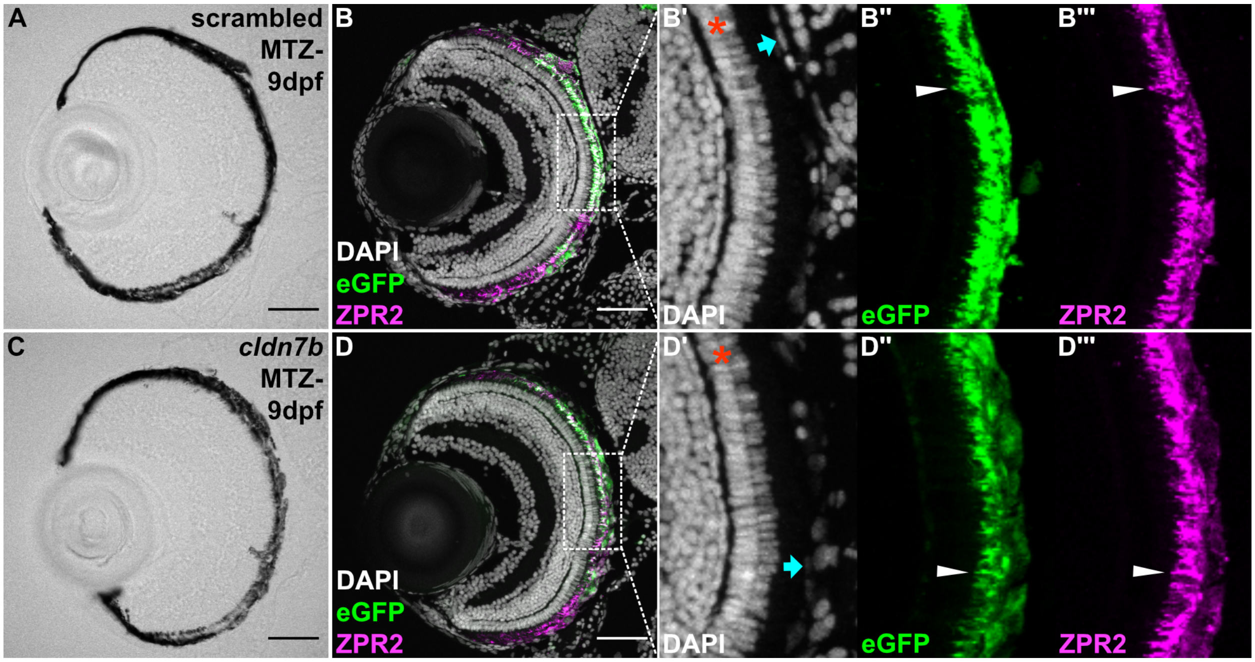
A
scrambled
MTZ-
9dpf
B
DAPI
eGFP
ZPR2
B'
DAPI
B''
eGFP
B'''
ZPR2
C
cldn7b
MTZ-
9dpf
D
DAPI
eGFP
ZPR2
D'
DAPI
D''
eGFP
D'''
ZPR2

FIGURE S5

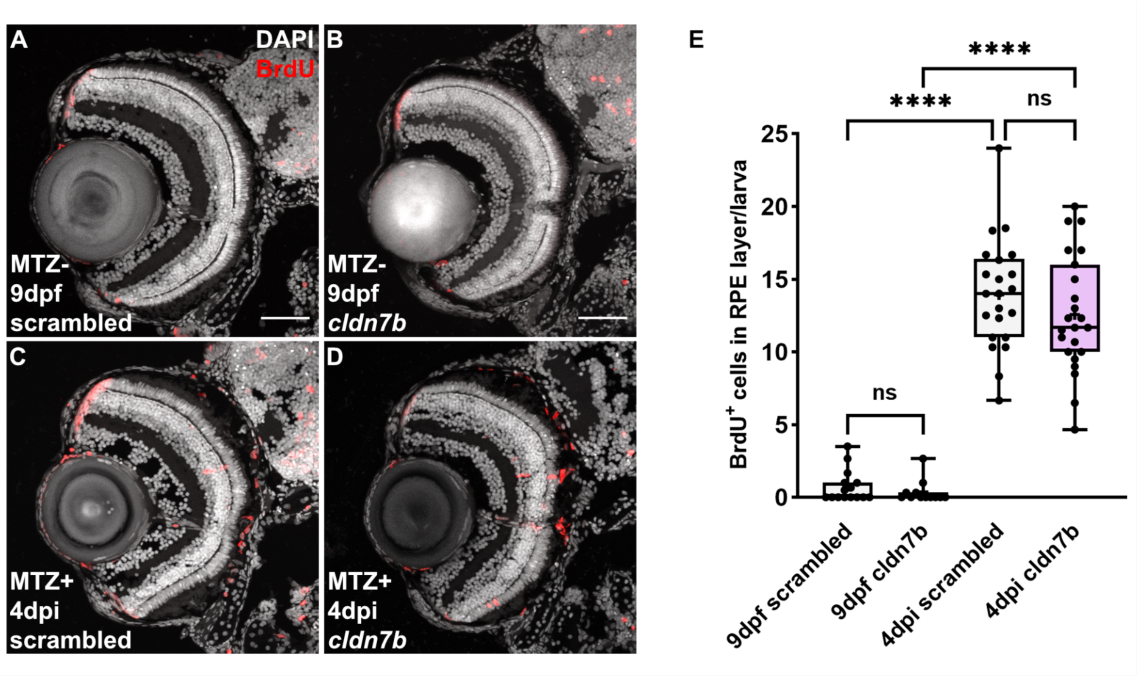
A
DAPI
BrdU
MTZ-
9dpf
scrambled
B
MTZ-
9dpf
cldn7b
C
MTZ+
4dpi
scrambled
D
MTZ+
4dpi
cldn7b
E
****
****
ns
ns
BrdU+ cells in RPE layer/larva
25
20
15
10
5
0
9dpf scrambled
9dpf cldn7b
4dpi scrambled
4dpi cldn7b

FIGURE S6

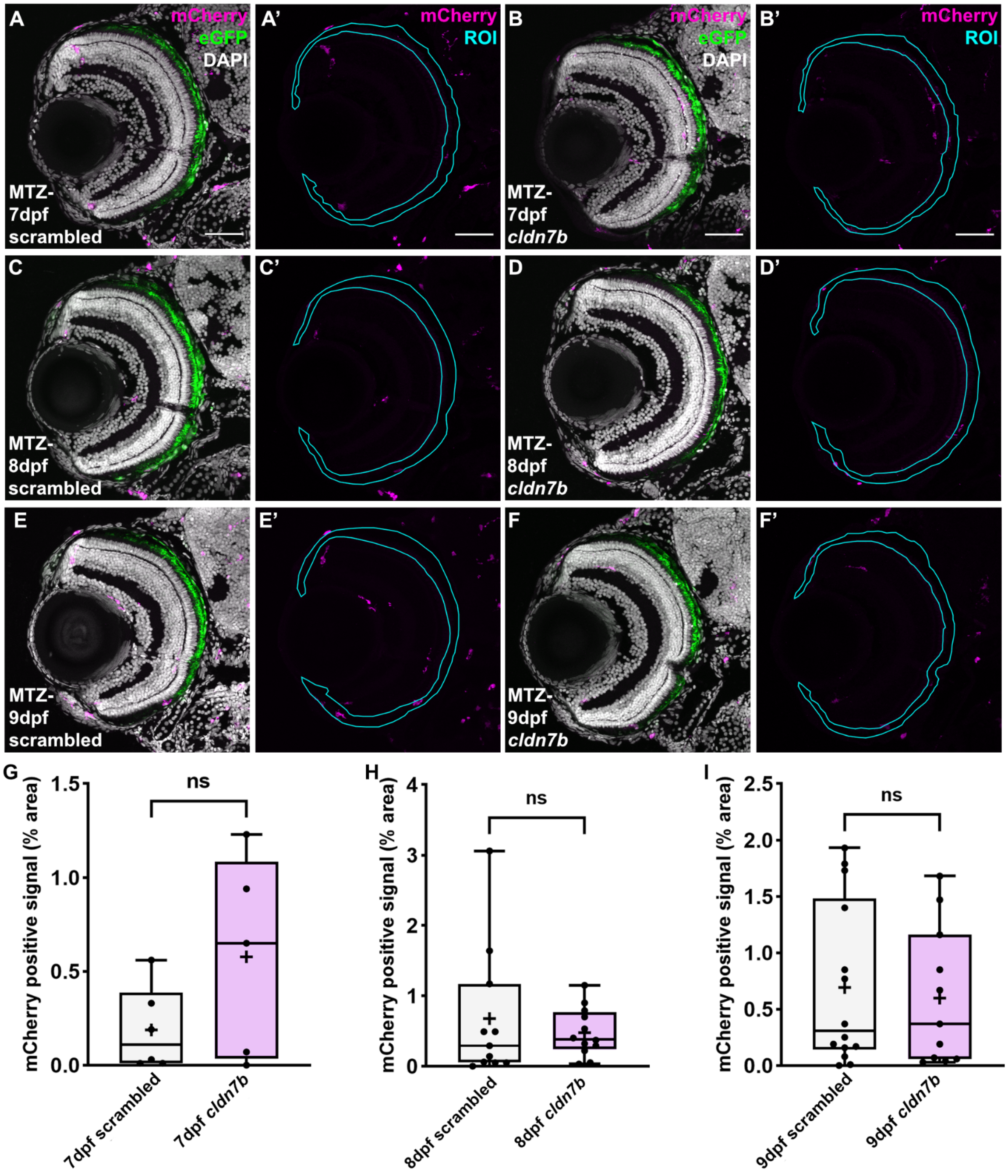
A
mCherry
eGFP
DAPI
MTZ-
7dpf
scrambled
A'
mCherry
ROI
B
mCherry
eGFP
DAPI
MTZ-
7dpf
cldn7b
B'
mCherry
ROI
C
MTZ-
8dpf
scrambled
C'
D
MTZ-
8dpf
cldn7b
D'
E
MTZ-
9dpf
scrambled
E'
F
MTZ-
9dpf
cldn7b
F'
G
mCherry positive signal (% area)
ns
7dpf scrambled
7dpf cldn7b
H
ns
8dpf scrambled
8dpf cldn7b
I
ns
9dpf scrambled
9dpf cldn7b

FIGURE S7

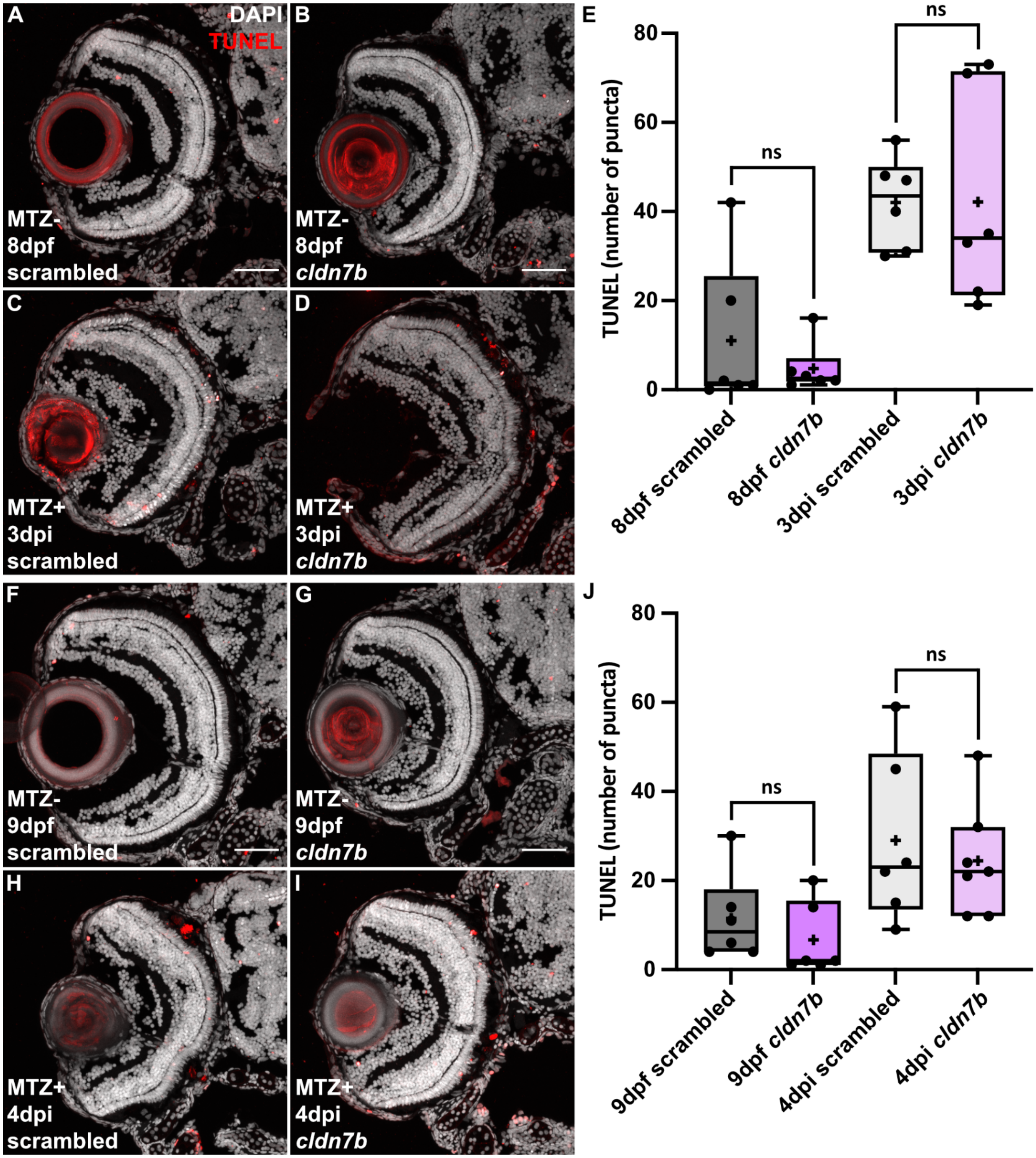

A
DAPI
TUNEL
MTZ-
8dpf
scrambled
B
MTZ-
8dpf
cldn7b
C
MTZ+
3dpi
scrambled
D
MTZ+
3dpi
cldn7b
E
TUNEL (number of puncta)
80
60
40
20
0
ns
ns
8dpf scrambled
8dpf cldn7b
3dpi scrambled
3dpi cldn7b
F
MTZ-
9dpf
scrambled
G
MTZ-
9dpf
cldn7b
H
MTZ+
4dpi
scrambled
I
MTZ+
4dpi
cldn7b
J
TUNEL (number of puncta)
80
60
40
20
0
ns
ns
9dpf scrambled
9dpf cldn7b
4dpi scrambled
4dpi cldn7b

FIGURE S8

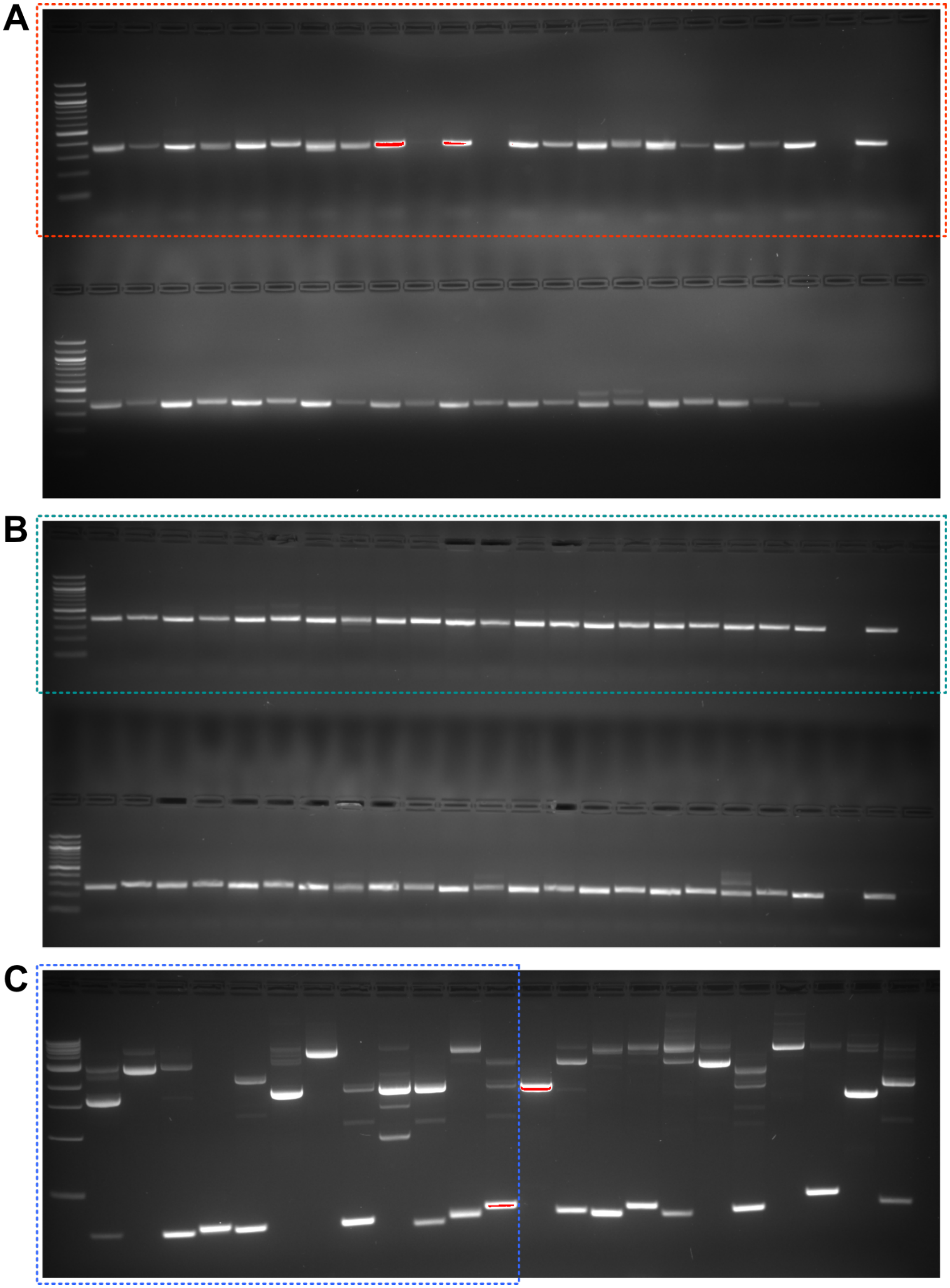

FIGURE S9

Gene Names	crRNA loci	custom crRNA design/names given by IDT predesigned crRNA database	crRNA Sequences	PAMs	Forward(F) Primers for headloop PCR	Reverse(R) Primers for headloop PCR	Amplicon siz	Tagged Primers (F/R) for headloop PCR		Mutagenic Rates(= H+ /S+ number of embryos)	F Primers for genotyping	R Primers for genotyping	Amplicon siz	Unvailbilit
	exon2		GGTTAGATACCACAAGATCC	TGG	GTGTGTTTGTATGTGTATCTGCAGGG	GGAGGAACATTCCATTGCAATTAGCC	288	F	**GGATCTTGTGGTATCTAACC**GTGTGTTTGTATGTGTATCTGCAGGG	7/10				
adamtsl7	exon3	custom	GGAGAATCCATCACTGCACC	TGG	GCTCTCAGCATGGCAACTGGTG	CGGCTGGCCATACACACAGACC	491	F	**GGTGCAGTGATGGATTCTCC**GCTCTCAGCATGGCAACTGGTG	10/10	N/A			29.2%
	exon7		GGCAAATCACCTGACGATAC	TGG	GCAAGCAGTAGTGCATGCCT	CACTGCAGATCTTGAGCTGGC	363	R	**GTATCGTCAGGTGATTTGCC**CACTGCAGATCTTGAGCTGGC	7/10				
	exon2	AA	GAAATCACGTTGCCTATAAC	CGG	GCTGCCATTGCTGTCATTATGTC	CCAGTGAATGCATTGTCACCCTC	353	F	**GTTATAGGCAACGTGATTTC**GCTGCCATTGCTGTCATTATGTC	10/10				
ccl34a.4	exon3	AB	GTTATAGGCAACGTGATTTC	TGG	CTCCATGCGTCAAAGCAGTCATG	CAGCGGTGTAGAAACAGTGCTG	322	F	**GCTCTGGTTCCAGTGAATGC**CTCCATGCGTCAAAGCAGTCATG	10/10	GCTGCCATTGCTGTCATTATGTC	CAAGCTGACTTATTTCAGCAGAACCC	696	18.7%
	exon4	custom	GCACTCTGTCTACACTTCTG	AGG	GGGAGAAGATCCAGGAGCTCATAATC	CAAGCTGACTTATTTCAGCAGAACCC	327	F	**CAGAAGTGTAGACAGAGTGC**GGGAGAAGATCCAGGAGCTCATAATC	10/10				
	exon2	AC	AAGGACTGCGCTGCCACCTA	GGG	GCTGAAATGTTCTTGTCCTTCCACTC	GTCCATTTTCTTTGCACTTTTCCCATG	499	F	**TAGGTGGCAGCGCAGTCCTT**GCTGAAATGTTCTTGTCCTTCCACTC	8/10				
ccn1l1	exon4	AB	GCTTGACACGCCTATTCCAC	AGG	GCACACTGTGCAAAAAGAGTCATG	GCTCTTACCTTCATTGATGGTGCAG	314	R	**GTGGAATAGGCGTGTCAAGC**GCTCTTACCTTCATTGATGGTGCAG	7/10	GCTGAAATGTTCTTGTCCTTCCACTC	GAAGAGTTCAGCTGGTGTGTTCAG	4393	36.9%
	exon5	custom	TGTGTGTCCCATCTGCAACT	CGG	CCACAGAAACCTGTGCAGATCAG	GAAGAGTTCAGCTGGTGTGTTCAG	246	F	**AGTTGCAGATGGGACACACA**CCACAGAAACCTGTGCAGA	9/10				
	exon3	AA	GTTGATGACACGGAAGGGTC	TGG	CTGGGATTTCAAGCCATTTTGCG	CAGTCTTCACCTTGTGGTGCAG	210	R	**GACCCTTCCGTGTCATCAAC**CAGTCTTCACCTTGTGGTGCAG	8/10				
cidec	exon4	AD	TGGAACTGGAATAGATACGC	AGG	GGCTCATTATTTTGCAATGTCGCTG	CTCCCAATGCGTCTGAGAATGTTTAC	497	F	**GCGTATCTATTCCAGTTCCA**GGCTCATTATTTTGCAATGTCGCTG	10/10	N/A			27.9%
	exon6	AB	GTTGCAATCAGGGTCTTTCT	TGG	CGAGTCGTTGACAATAGCAGTGTG	GTACATTCCATACAAGGTTGCCTGC	243	R	**AGAAAGACCCTGATTGCAAC**GTACATTCCATACAAGGTTGCCTGC	10/10				
	exon2		ATACGAGCTAACCAAATACC	TGG	GTGTGACCGTTCATTTTTGGCTG	CGTCATCCTCACACGCTGATAAC	287	F	**GGTATTTGGTTAGCTCGTAT**GTGTGACCGTTCATTTTTGGCTG	10/10				
clcf1	exon3	custom	CTATCCCATCGTCTCACTCT	GGG	CCAAAGAAGAGGGAGAGAAGGAGG	CACCTTCCGCGAGAAGTCATTTG	332	F	**AGAGTGAGACGATGGGATAG**CCAAAGAAGAGGGAGAGAAGGAGG	9/9	GTGTGACCGTTCATTTTTGGCTG	CACCTTCCGCGAGAAGTCATTTG	2857	24.9%
	exon3		ACACGTGTGGACTTATGGCG	CGG	CAATAAGGATGCACAGTGGATGGC	CCAGATATTGAGCCAAGCAACCC	416	F	**CGCCATAAGTCCACACGTGT**CAATAAGGATGCACAGTGGATGGC	10/10				
	exon1	AB	CGCACGACATCCAAAGGCCC	TGG	CGCACGACATCCAAAGGCCC	GTGAATAGTACGCGCATGCGTG	397	R	**GGGCCTTTGGATGTCGTGCG**GTGAATAGTACGCGCATGCGTG	10/10				
cldn7b	exon3	AG	TTGACTTGTAAACCAGCCAC	AGG	CTCCTGTTGTGCGCTGAAAAATGTC	GTTCATCCCAGCCTGTAGGTCATTG	432	R	**GTGGCTGGTTTACAAGTCAA**GTTCATCCCAGCCTGTAGGTCATTG	8/8	CGCACGACATCCAAAGGCCC	CCAGGTCGTGAACAAATGGCTC	4251	17.0%
	exon4	custom	ATCGAGAAATGCACCAGCCC	AGG	CAGCTAGCAACAACACTGTGAAGTG	CCAGGTCGTGAACAAATGGCTC	354	R	**GGGCTGGTGCATTTCTCGAT**CCAGGTCGTGAACAAATGGCTC	10/10				
	exon5	AB	GTACATGGGACGGTTCTCGT	AGG	CGCCAAGATCTTCGACTTTGCTG	CACCCGTGCTGAACTGGAATC	324	R	**ACGAGAACCGTCCCATGTAC**CACCCGTGCTGAACTGGAATC	8/8				
cpa4	exon6	AA	CACGGGTGGTGAGAACCGAC	CGG	GGACACCCTTGTTGCAAGTCAC	GAGGGATCGACGCCATAGTCTAAAG	374	F	**GTCGGTTCTCACCACCCGTG**GGACACCCTTGTTGCAAGTCAC	8/9	CGCCAAGATCTTCGACTTTGCTG	CAATCTAAAGACAGCCGTGATTGTTGC	658	23.0%
	exon7	AC	CCCTCCGTGACCTCTCTTCT	GGG	CAGGCTTCTGCTGTTTGGATCG	CAATCTAAAGACAGCCGTGATTGTTGC	275	F	**AGAAGAGAGGTCACGGAGGG**CAGGCTTCTGCTGTTTGGATCG	9/10				
	exon1	AB	AGTGTGAACAGACTGAGAGC	AGG	CAAGGAGGACAGTTCAGTGCAC	CTCGGATGAATGTTGCAGCTGTC	259	R	**GCTCTCAGTCTGTTCACACT**CTCGGATGAATGTTGCAGCTGTC	10/10				
cxcl18a.1	exon2	AA	ATTTGAACCTTGTTGCAAAG	AGG	CTGCCAGATACTGGTCATGGAG	GACATGAGGGTGACGGATGATG	593	R	**CTTTGCAACAAGGTTCAAAT**GACATGAGGGTGACGGATGATG	10/10	CAAGGAGGACAGTTCAGTGCAC	CTGCAGAGTATCTGCTGATTTCTGC	1311	35.0%
	exon3	AC	ACACTTCTTTATTTGACAAC	TGG	GTATCATTGCTCGCAAAGTAGTGCG	CTGCAGAGTATCTGCTGATTTCTGC	267	R	**GTTGTCAAATAAAGAAGTGT**CTGCAGAGTATCTGCTGATTTCTGC	10/10				
	exon2		TTATGTGTTTTCCAATGCGT	CGG	CTGGCATTTCTGACCATCATTGAAGG	GTTGTCATCAAGGTGGCACTGTG	400	R	**ACGCATTGGAAAACACATAA**GTTGTCATCAAGGTGGCACTGTG	10/10				
cxcl8a	exon3	custom	TCTGGACCCCTCTGCTCCAT	GGG	GAAACAGAAAGCCGACGCATTGG	CGGGCATTCATGGTTTTCTGCTG	414	F	**ATGGAGCAGAGGGGTCCAGA**GAAACAGAAAGCCGACGCATTGG	10/10	CTGGCATTTCTGACCATCATTGAAGG	CGGGCATTCATGGTTTTCTGCTG	598	16.2%
	exon3		CTGTCCAGTTGTCATCAAGG	TGG	GAAACAGAAAGCCGACGCATTGG	CGGGCATTCATGGTTTTCTGCTG	414	R	**CCTTGATGACAACTGGACAG**CGGGCATTCATGGTTTTCTGCTG	10/10				
	exon2	AF	TTTGTCTGTCGTGCCGCAAG	CGG	GGTTAACCAGGTGGTTATGATGCCTG	GGAGAAATGAACCTTCATGCATGCTG	283	F	**CTTGCGGCACGACAGACAAA**GGTTAACCAGGTGGTTATGATGCCTG	10/10				
dkk1a	exon3	AB	CTGGTGTGGCTGTAACACGT	GGG	CCGATGCATCAACGGTACAAACG	GGAGATACGCAAGGAGAAACATGACAG	361	F	**ACGTGTTACAGCCACACCAG**CCGATGCATCAACGGTACAAACG	10/10	GGTTAACCAGGTGGTTATGATGCCTGGT	GTTTTTCTCTCTGGCCTCTGCAGG	2507	21.9%
	exon4	custom	CTGGTCACGGATCTGTAAGC	CGG	CCAGCACGTCTCTGAATGCAGATC	GTTTTTCTCTCTGGCCTCTGCAGG	260	F	**GCTTACAGATCCGTGACCAG**CCAGCACGTCTCTGAATGCAGATC	10/10				
	exon2	AD	ATTATCTAACCAGCTACTAC	AGG	GTTGGCAAATGTAGATTCCAGCACAAG	GCAGGTTTGGAACAAATGAAAGGAG	436	R	**GTAGTAGCTGGTTAGATAAT**GCAGGTTTGGAACAAATGAAAGGAG	10/10				
edn2	exon3	AA	ACTGGAGCAGGTACGATCGG	CGG	CCTTTGTACAACAGTAAGATCACCCCG	GCCAATAGACAGTTTCTCTTTCTCTCCC	324	R	**CCGATCGTACCTGCTCCAGT**GCCAATAGACAGTTTCTCTTTCTCTCCC	8/8	GTTGGCAAATGTAGATTCCAGCACAAGG	GATTTGATCTTCAGGGTGGAGCG	870	33.0%
	exon3	AB	AAGATCACCCCGCTTGGTCT	TGG	CTCCTTTCATTTGTTCCAAACCTGC	GATTTGATCTTCAGGGTGGAGCG	459	F	**AGACCAAGCGGGGTGATCTT**CTCCTTTCATTTGTTCCAAACCTGC	10/10				
	exon3	AA	TCCCACCCGATCTTGTGTCC	TGG	CACGAGCACAGACATGCCTAAC	CACTGTTGACGCACCAGCATAC	302	R	**GGACACAAGATCGGGTGGGA**CACTGTTGACGCACCAGCATAC	10/10				
epcam	exon4	AC	CTCAGTTTGGTGGCATCAAT	GGG	GAACATAAAGTGCGAGCCTGCG	CCAAACCGTAACGCTGCTGTAG	365	R	**ATTGATGCCACCAAACTGAG**CCAAACCGTAACGCTGCTGTAG	10/10	CACGAGCACAGACATGCCTAAC	CAGCAATGATACCACCAGTCAGC	1152	16.9%
	exon7	custom	CCCGGAACATCAACCTCAAA	TGG	CGACCGTACTACAGATCTGTCCC	CAGCAATGATACCACCAGTCAGC	293	R	**TTTGAGGTTGATGTTCCGGG**CAGCAATGATACCACCAGTCAGC	10/10				
	exon2	AD	TAACCAACCTAATTCGCTCC	CGG	CGAAAGAACGTAAGTACACACCGTG	CAACGACACTGAACTGAAAGGACAC	640	R	**GGAGCGAATTAGGTTGGTTA**CAACGACACTGAACTGAAAGGACAC	9/9				
epha2a	exon3	AA	CCGCAAGAGCCAGTTTCGAA	AGG	CTGTAGAGCTGAGCTTCATCGTCC	GACAGGTGGTGTAGAACACCCTC	321	F	**TTCGAAACTGGCTCTTGCGG**CTGTAGAGCTGAGCTTCATCGTCC	10/10	N/A			20.3%
	exon4	custom	AACACCCAACGTTCGGGACG	GGG	GGTCTGAAACGAGTAAAGGGTTCAGC	GGAAGAGCTAGAGAAGCAGAGCAGTG	396	F	**CGTCCCGAACGTTGGGTGTT**GGTCTGAAACGAGTAAAGGGTTCAGC	7/7				
	exon1		GAGAGTCGTATTATGATTCC	CGG	GGCGTAAGGACACTGAAGAAAAGC	CGTAGAGTCCTCAGTGTCATTCTGC	506	F	**GGAATCATAATACGACTCTC**GGCGTAAGGACACTGAAGAAAAGC	10/10				
fosl1b	exon2	custom	CCACTGCTCCATTACAGGAC	GGG	GCAGAATGACACTGAGGACTCTACG	CTACAGCACTGACTCACTCCTCC	410	R	**GTCCTGTAATGGAGCAGTGG**CTACAGCACTGACTCACTCCTCC	10/10	GGCGTAAGGACACTGAAGAAAAGC	GATCTGAACCAGCAGATTCAGCAC	3635	35.6%
	exon4		AAGAGATTGCCACACTTGAA	AGG	GAAAGGAACAGGGTAGCAGCTG	GATCTGAACCAGCAGATTCAGCAC	330	F	**TTCAAGTGTGGCAATCTCTT**GAAAGGAACAGGGTAGCAGCTG	10/10				
	exon2		TTGCTGGAAGAGACTTGAAC	CGG	CTGGTGGATGCCAGCAGTTTC	GGGTCTGATTGCAGCAAGACC	367	R	**GTTCAAGTCTCTTCCAGCAA**GGGTCTGATTGCAGCAAGACC	9/9				
il11a	exon3	custom	GACATGGTCTCCCACATCGG	GGG	CTCTCATCGCAGGTCAAGCCTAC	CTTGCCTGATCACCCTAACCATCC	501	F	**CCGATGTGGGAGACCATGTC**CTCTCATCGCAGGTCAAGCCTAC	10/10	CTGGTGGATGCCAGCAGTTTC	CCGGACTGACGATACAACTCATC	2833	32.0%
	exon4		CGTCCTGCCCAACACTCGAG	CGG	GACATAGTGTGAAAATGCCCTTGC	CCGGACTGACGATACAACTCATC	462	R	**CTCGAGTGTTGGGCAGGACG**CCGGACTGACGATACAACTCATC	6/8				
	exon1		CAAGCCATCAGGATGATGAG	GGG	GTTTTTGTGCTAACAGTGTCGCC	TGGGAAAACTAGCCCTCAAAATGAC	284	R	**CTCATCATCCTGATGGCTTG**TGGGAAAACTAGCCCTCAAAATGAC	9/10				
il11b	exon3	custom	CCAATTCGTCACTATTCCGT	TGG	CCTACCAATATGCCATAGTGGGTC	GTAGGTGACAGACAGCACAGTTG	486	R	**ACGGAATAGTGACGAATTGG**GTAGGTGACAGACAGCACAGTTG	6/8	GTTTTTGTGCTAACAGTGTCGCC	GCACCATAAAGATTGGCTGACGG	2375	30.1%
	exon4		CTTCAGGGATCCAAGTGCTC	TGG	CTTACAGCTCGGACAGACAGCA	GCACCATAAAGATTGGCTGACGG	212	R	**GAGCACTTGGATCCCTGAAG**GCACCATAAAGATTGGCTGACGG	6/8				
	exon1	AA	GACCCACGGCTCCCGAAGAC	AGG	GCTTGTTAATATCATCCCTGGTGGCC	GAAGTACATCATGCTAAACCAAGTGCCAAC	397	F	**GTCTTCGGGAGCCGTGGGTC**GCTTGTTAATATCATCCCTGGTGGCC	7/10				
lepb	exon2	AB	GGAGGAACTGGCCGTCTCAC	AGG	GTCTCCAGAGATTGATTTCGGCCC	GTTGAGGCAGAGCTTCTCCAGG	301	F	**GTGAGACGGCCAGTTCCTCC**GTCTCCAGAGATTGATTTCGGCCC	10/10	GCTTGTTAATATCATCCCTGGTGGCC	GTTGAGGCAGAGCTTCTCCAGG	1877	36.7%
	exon2	AD	GACATCGACAACCCCATTGA	TGG	GGGAAAAAAGGCTCTTGGTTCTTGGC	CCTCCAGTGTCCTCAGGAGAGTCTC	383	F	**TCAATGGGGTTGTCGATGTC**GGGAAAAAAGGCTCTTGGTTCTTGGC	6/6				
	exon2		GACCACACTCGAGGTTGGCA	CGG	GTGCACACCTGTAAGCAAGTGTC	CGATGTGGTTGATCCAGATTGGC	269	R	**TGCCAACCTCGAGTGTGGTC**CGATGTGGTTGATCCAGATTGGC	10/10				
lipib	exon4	custom	GTTGGATCGAGACGTTCCTC	AGG	GGCACAATGCCTTCCTCAGTTAG	GTGGGTGTCTCCTGTATTGTTTTTCC	286	R	**GAGGAACGTCTCGATCCAAC**GTGGGTGTCTCCTGTATTGTTTTTCC	10/10	GTGCACACCTGTAAGCAAGTGTC	GTATCTCCACCCTGACCTCTCC	5080	40.5%
	exon5		CGGCTGATCTAATCCTCCAT	TGG	CCTATGCTCTGTTTCCTGTGGC	CCTATGCTCTGTTTCCTGTGGC	162	R	**ATGGAGGATTAGATCAGCCG**GTATCTCCACCCTGACCTCTCC	10/10				
	exon2	AA	TCGAGTTGCCACCTTCGCGT	TGG	CGTTGCGAACTCTGAACCTCTTGAG	GTTGAAAACCACTTGAGCCGAACG	358	R	**ACGCGAAGGTGGCAACTCGA**GTTGAAAACCACTTGAGCCGAACG	10/10				
met	exon2	AB	TAAGACTGGACCGGTCCATG	AGG	CTCCAAACTCGACCTCTCAGTGAC	CCTCTAACACATGACGACTGCAGAC	341	F	**CATGGACCGGTCCAGTCTTA**CTCCAAACTCGACCTCTCAGTGAC	10/10	N/A			20.7%
	exon11	custom	CGTGGAAGATCCCAAATTTG	AGG	GACCTGCAGTCATGATGAGGATAAG	TGACACTATGCGTGACAATGCATAC	317	F	**CAAATTTGGGATCTTCCACG**GACCTGCAGTCATGATGAGGATAAG	9/10				
	exon1	AB	TCCTCTGTCACCTGTAATGA	CGG	GAGTTTTCCTCTGGACTTCACAGC	GGAGTGAAGTTCGAACCTTCAGC	264	F	**TCATTACAGGTGACAGAGGA**GAGTTTTCCTCTGGACTTCACAGC	7/7				
notum1b	exon2	AA	GATTCTGTCTCCACAACCAG	AGG	GACCAACCCAGTACTAGTAAGGTG	CCCATGAACGCATAGTCATCTGAAC	563	F	**CTGGTTGTGGAGACAGAATC**GACCAACCCAGTACTAGTAAGGTG	8/10	GAGTTTTCCTCTGGACTTCACAGC	CTCCTTGATGACCTCCTGAATGATC	4259	32.3%
	exon3	custom	CAAACGTCACTGGAGCAGTA	CGG	CTGAGACAACATATGCTGGATATGGG	CTCCTTGATGACCTCCTGAATGATC	343	R	**TACTGCTCCAGTGACGTTTG**CTCCTTGATGACCTCCTGAATGATC	8/10				
	exon2		CCATGGCAAGTGCTTCACGC	TGG	GCTCTGCAGTAAAACCTCTCCTC	CATGTAGAACGACAGTGTGACGG	276	F	**GCGTGAAGCACTTGCCATGG**GCTCTGCAGTAAAACCTCTCCTC	10/10				
nrg1	exon5	custom	TCCACCGGAAAACCTGCAGC	TGG	GCAATGTCCTCCCGTCTAAGAG	GTAAGCCGTCAGCTAGTAAGCG	382	F	**GCTGCAGGTTTTCCGGTGGA**GCAATGTCCTCCCGTCTAAGAG	10/10	N/A			30.3%
	exon6		TCTGTCATATGAGCTGGGAC	AGG	GCCCCATTTTGGTGTCTTTGTC	GGCTAGATGAGTGAGTGATGGC	277	R	**GTCCCAGCTCATATGACAGA**GGCTAGATGAGTGAGTGATGGC	10/10				
	exon2	AB	ACATGATGGAGTCCTCCACC	AGG	CGTCAGTGTTTCAACATCACAAGAGG	GAATGTGTTTCTGCTGACTGTACAGC	413	F	**GGTGGAGGACTCCATCATGT**CGTCAGTGTTTCAACATCACAAGAGG	9/10				
ogfrl1	exon3	AA	CGATATTCATTCGGCTGACG	AGG	GCTGTACAGTCAGCAGAAACACATTC	GCAGCTTTGTACTGTGAAAAGACTTCC	337	R	**CGTCAGCCGAATGAATATCG**GCAGCTTTGTACTGTGAAAAGACTTCC	10/10	N/A			13.3%
	exon7	AD	ACTCGCATTCTGAAGAGTCT	TGG	GCGTCCTTTCATGTTTGGCATTG	GTGTGAAGACGAAGTATTCCAGTGC	261	F	**AGACTCTTCAGAATGCGAGT**GCGTCCTTTCATGTTTGGCATTG	10/10				
	exon4	AA	CCGACGTACAATGCTGATTA	TGG	CAGCATCTCTTACCTGAGGGATGG	CATGAGACTGCTCCTCTGTGGATC	373	F	**TAATCAGCATTGTACGTCGG**CAGCATCTCTTACCTGAGGGATGG	7/10				
ptgs2a	exon2	AB	CCTTGGATAAACACACGCCC	TGG	CTCGACCAGAGTAACGCATCTCTG	GCGTGTGAGAAGCTCAGCTGTAAG	452	R	**GGGCGTGTGTTTATCCAAGG**GCGTGTGAGAAGCTCAGCTGTAAG	10/10	CTCGACCAGAGTAACGCATCTCTG	CGTCAACGTATAAATCGCACCCTG	1237	28.4%
	exon5	AF	GCATATTTATGGAGAGACGC	TGG	CTCTCACCAGTTCTTCAAGTCGGA	CGTCAACGTATAAATCGCACCCTG	309	F	**GCGTCTCTCCATAAATATGC**CTCTCACCAGTTCTTCAAGTCGGA	9/9				
	exon4	AA	ATTGGCGTTGGGCAATCACG	CGG	GCCCATCTGATCGATAGTCCTCC	GCTTATCCACACACCCAAGGCTC	256	R	**CGTGATTGCCCAACGCCAAT**GCTTATCCACACACCCAAGGCTC	10/10				
ptgs2b	exon2	AB	CGAGGTGTCTGTACGGAAAT	GGG	GGTTTGCACAGGAGGTAAGCCTAC	GCCAAAAGCAGAAATGGGCAGAG	328	F	**ATTTCCGTACAGACACCTCG**GGTTTGCACAGGAGGTAAGCCTAC	10/10	GGTTTGCACAGGAGGTAAGCCTAC	CAAATACGCACCCCATGGTTCAG	2344	24.4%
	exon5	AD	AGATTGGTGCGTTGAGGATC	TGG	CCTTGGGTGTGTGGATAAGCAAC	CAAATACGCACCCCATGGTTCAG	420	R	**GATCCTCAACGCACCAATCT**CAAATACGCACCCCATGGTTCAG	10/10				
	exon1		AATACCTGATGAGGACCAGT	TGG	CTCATATTAACACATGGCCAGCAAGTC	GTCACTTCATCGATTTCTGTTTGGCAG	417	F	**ACTGGTCCTCATCAGGTATT**CTCATATTAACACATGGCCAGCAAGTC	10/10				
ptx3a	exon2	custom	TGTGAAGAGCTTATCCCATT	TGG	GTGGATAGCATGTTGACGCAATGAC	CAGATGCGCAAGATCCACCATAC	435	R	**AATGGGATAAGCTCTTCACA**CAGATGCGCAAGATCCACCATAC	10/10	N/A			31.8%
	exon3		ATTGGTCCTAAGCAATCCAT	GGG	GCCTGACACTTCATTCAAGTCTGG	GATGCACTCTGTCCACTCAACAC	307	R	**ATGGATTGCTTAGGACCAAT**GATGCACTCTGTCCACTCAACAC	10/10				
	exon1	AC	CAGAGAGTAGCCCATCTTGG	AGG	CAGTCTGCTCCGGATCGAAATCTG	GCTACCTCCACTCCATCTTCACTG	308	R	**CCAAGATGGGCTACTCTCTG**GCTACCTCCACTCCATCTTCACTG	10/10				
serpine1	exon2	AD	GGTTGACCGGAAGATCATCT	TGG	GGTTGACCGGAAGATCATCT	CATCAGTGTGGTCAGACGTCCAG	379	F	**AGATGATCTTCCGGTCAACC**GCTACTCTAAAACTGCTCGCCTCC	9/10	CAGTCTGCTCCGGATCGAAATCTG	GCCATCCTTAGACACGAACTCACC	903	23.5%
	exon3	AB	GAATGCCCTGCACTTCCACG	GGG	CTGGACGTCTGACCACACTGATG	GCCATCCTTAGACACGAACTCACC	463	F	**CGTGGAAGTGCAGGGCATTC**CTGGACGTCTGACCACACTGATG	10/10				
	exon2	AA	ATTCCCTGTGGATTCTTCGT	GGG	CTGCTGCCAACAAACTGATCATTC	CTGCTGCCAACAAACTGATCATTC	305	F	**ACGAAGAATCCACAGGGAAT**CTGCTGCCAACAAACTGATCATTC	10/10				
zgc:153911	exon3	AC	GACGGATGGAGTCAATCTGC	TGG	ACCTGCTCCATCAGCACAAACTC	CAGCTGGACACGATGTAAAGTCC	350	F	**GCAGATTGACTCCATCCGTC**ACCTGCTCCATCAGCACAAACTC	8/10	CTGCTGCCAACAAACTGATCATTC	CTGTGACAAACACACCAGATATCTGAC	1368	33.3%
	exon4	custom	CAGACTTTTAGTTTCACTGG	AGG	CAGACTTTTAGTTTCACTGG	CTGTGACAAACACACCAGATATCTGAC	358	R	**CCAGTGAAACTAAAAGTCTG**CTGTGACAAACACACCAGATATCTGAC	9/10				
	N/A	#1	CGTTAATCGCGTATAATACG											
**scrambled**	N/A	#2	CATATTGCGCGTATAGTCGC	NA										6.0%
	N/A	#3	GGCGCGTATAGTCGCGCGTA											

Table S1. crRNAs and primers used in this study.

Table S2. RPE regions (>20 continuous angular degrees) with significant differences.

Gene name	Region(s) with significant difference (>20 angular degrees) compared to scrambled controls	Total region size (angular degrees)
nrg1	40-63(+); 81-143(+)	85
fosl1b	34-67(+); 105-150(+)	78
ogflr1	30-62(+); 103-134(+)	63
ccn1l1	100-150(+)	50
lipib	104-150(+)	46
cldn7b	101-133(+)	32
cidec	101-131(+)	30
il11a	104-126(+)	22
zgc:153911	30-111(-)	81
cpa4	30-106(-)	76
adamtsl7	55-82(-); 104-147(-)	70
dkk1a	30-76(-)	46
epha2a	82-102(-); 120-143(-)	43
lepb	37-71(-)	34
serpine1	61-91(-)	30

(+)/(-) indicate higher/lighter (+) or lower/darker (-) pixel intensity in the F0 knockouts than the scrambled controls.

Table S3. Statistics

Figures	Numbers of independent experiments (N)	Compared groups	Statistical tests	Biological replicates (n)	*p*-values
Fig. 3G	N=1, N=1	2dpi scrambled vs. 2dpi *cldn7b*	unpaired Student's t-test with Welch's correction	n=9, n=11	*p*=0.2367
	N=3, N=3	3dpi scrambled vs. 3dpi *cldn7b*	unpaired Student's t-test with Welch's correction	n=19, n=23	*p*=0.0017
	N=3, N=3	4dpi scrambled vs. 4dpi *cldn7b*	unpaired Student's t-test with Welch's correction	n=14, n=16	*p*=0.0040
Fig. S6E	N=3, N=3, N=3, N=3	9dpf scrambled vs. 9dpf *cldn7b* vs. 4dpi scrambled vs. 4dpi scrambled	Kruskal–Wallis one-way ANOVA	n=15, n=12, n=22, n=23	H=51.29 *p*<0.0001
		9dpf scrambled vs. 9dpf *cldn7b*	Dunn's multiple comparison test		*p*>0.9999
		4dpi scrambled vs. 4dpi *cldn7b*	Dunn's multiple comparison test		*p*>0.9999
		9dpf scrambled vs. 4dpi scrambled	Dunn's multiple comparison test		*p*<0.0001
		9dpf *cldn7b* vs. 4dpi *cldn7b*	Dunn's multiple comparison test		*p*<0.0001
Fig. S7G	N=1, N=1	7dpf scrambled vs. 7dpf *cldn7b*	Mann Whitney test	n=6, n=5	*p*=0.3030
Fig. S7H	N=3, N=3	8dpf scrambled vs. 8dpf *cldn7b*	Mann Whitney test	n=11, n=12	*p*=0.7509
Fig. S7I	N=3, N=3	9dpf scrambled vs. 9dpf *cldn7b*	unpaired Student's t-test with Welch's correction	n=14, n=11	*p*=0.7285
Fig. S8E	N=1, N=1	8dpf scrambled vs. 8dpf *cldn7b*	Mann Whitney test	n=6, n=6	*p*=0.6970
	N=1, N=1	3dpi scrambled vs. 3dpi *cldn7b*	Mann Whitney test	n=6, n=6	*p*=0.8182
Fig. S8J	N=1, N=1	9dpf scrambled vs. 9dpf *cldn7b*	Mann Whitney test	n=6, n=6	*p*=0.1861
	N=1, N=1	4dpi scrambled vs. 4dpi *cldn7b*	Mann Whitney test	n=6, n=7	*p*=0.8065
